# Supplementary material for: Establishment of Wolbachia Strain wAlbB in Malaysian Populations of Aedes aegypti for Dengue Control
Source: Curr Biol. 2019 Dec 16;29(24):4241–4248.e5. doi: 10.1016/j.cub.2019.11.007 (PMC6926472; doi:10.1016/j.cub.2019.11.007)
Supplement: Document S1. Figures S1–S7 [file mmc1.pdf]

Current Biology, Volume *29*

Supplemental Information

Establishment of *Wolbachia* Strain *w*AlbB in Malaysian Populations of *Aedes aegypti* for Dengue Control

Wasi A. Nazni, Ary A. Hoffmann, Ahmad NoorAfizah, Yoon Ling Cheong, Maria V. Mancini, Nicholas Golding, Ghazali M.R. Kamarul, Mohd A.K. Arif, Hasim Thohir, Halim NurSyamimi, M. Zabari ZatilAqmar, Mazni NurRuqqayah, Amran NorSyazwani, Azmi Faiz, Francis-Rudin M.N. Irfan, Subramaniam Rubaaini, Nasir Nuradila, Nasir M.N. Nizam, Saidin M. Irwan, Nancy M. Endersby-Harshman, Vanessa L. White, Thomas H. Ant, Christie S. Herd, Asim H. Hasnor, Rahman AbuBakar, Dusa M. Hapsah, Khairuddin Khadijah, Denim Kamilan, Soo Cheng Lee, Yusof M. Paid, Kamaludin Fadzilah, Omar Topek, Balvinder S. Gill, Han Lim Lee, and Steven P. Sinkins

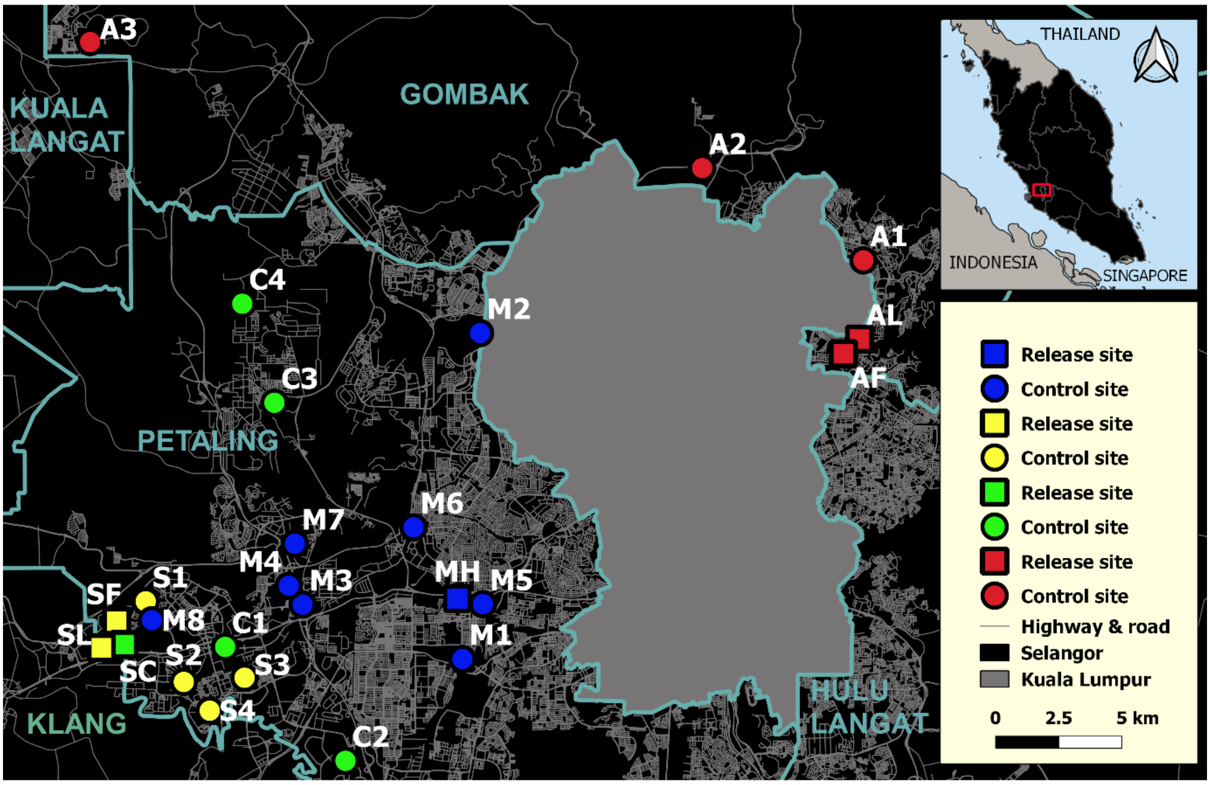

Figure S1. Maps of all six release zones and controls in the Petaling and Gombak Districts of Selangor, Related to Figure 1. Mentari Court high rise (MH) and controls (M1-M8); S7 Flats & Landed (SF & SL) and controls (S1-S4); S7 Commercial (SC) and controls (C1-C4); AU2 landed & AU2 PKNS Flats (AL & AF) and controls (A1-3). Table 1 contains additional details about release and controls sites. Map constructed using MapCruzin.com, OpenStreetMap.org under the Open Database License (http://opendatacommons.org/licenses/odbl/1.0/)).

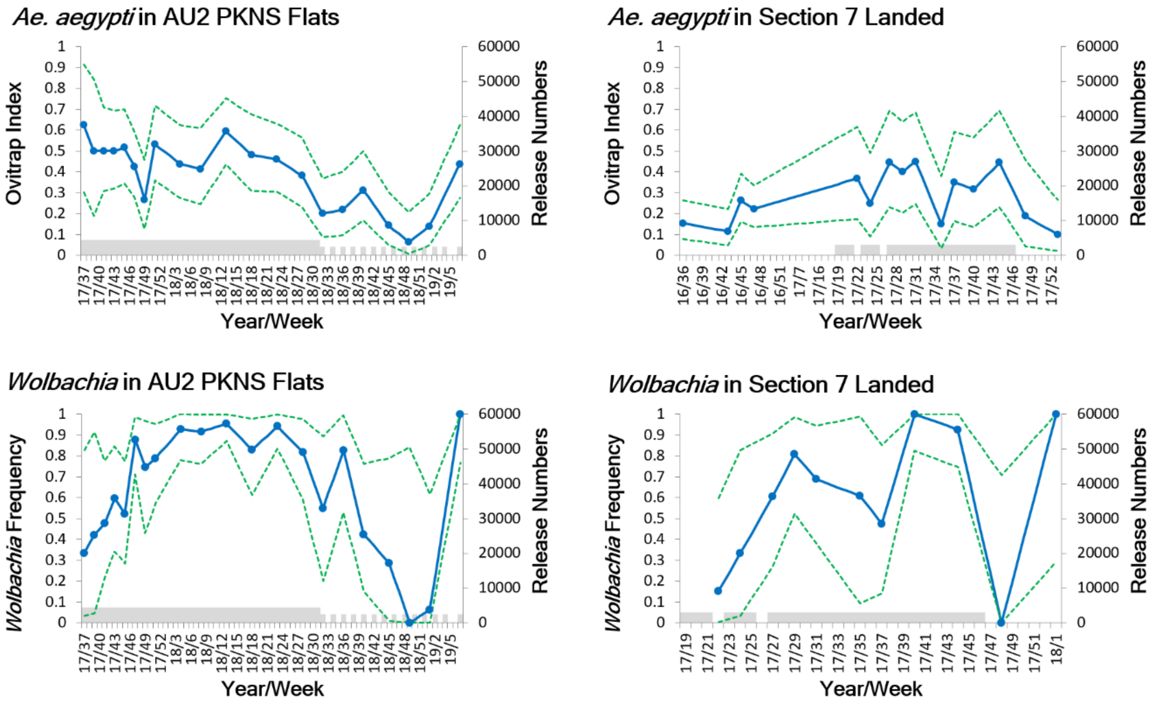

Figure S2. ***Aedes aegypti* population size and *Wolbachia* frequency estimates for two smaller secondary release sites, Related to Figures 2 and 3.** Population size was measured through an ovitrap index (*Ae. aegypti*- positive traps divided by total number of traps) during the release/monitoring period. Grey shaded areas represent release periods; 95% confidence intervals are shown as dotted lines.

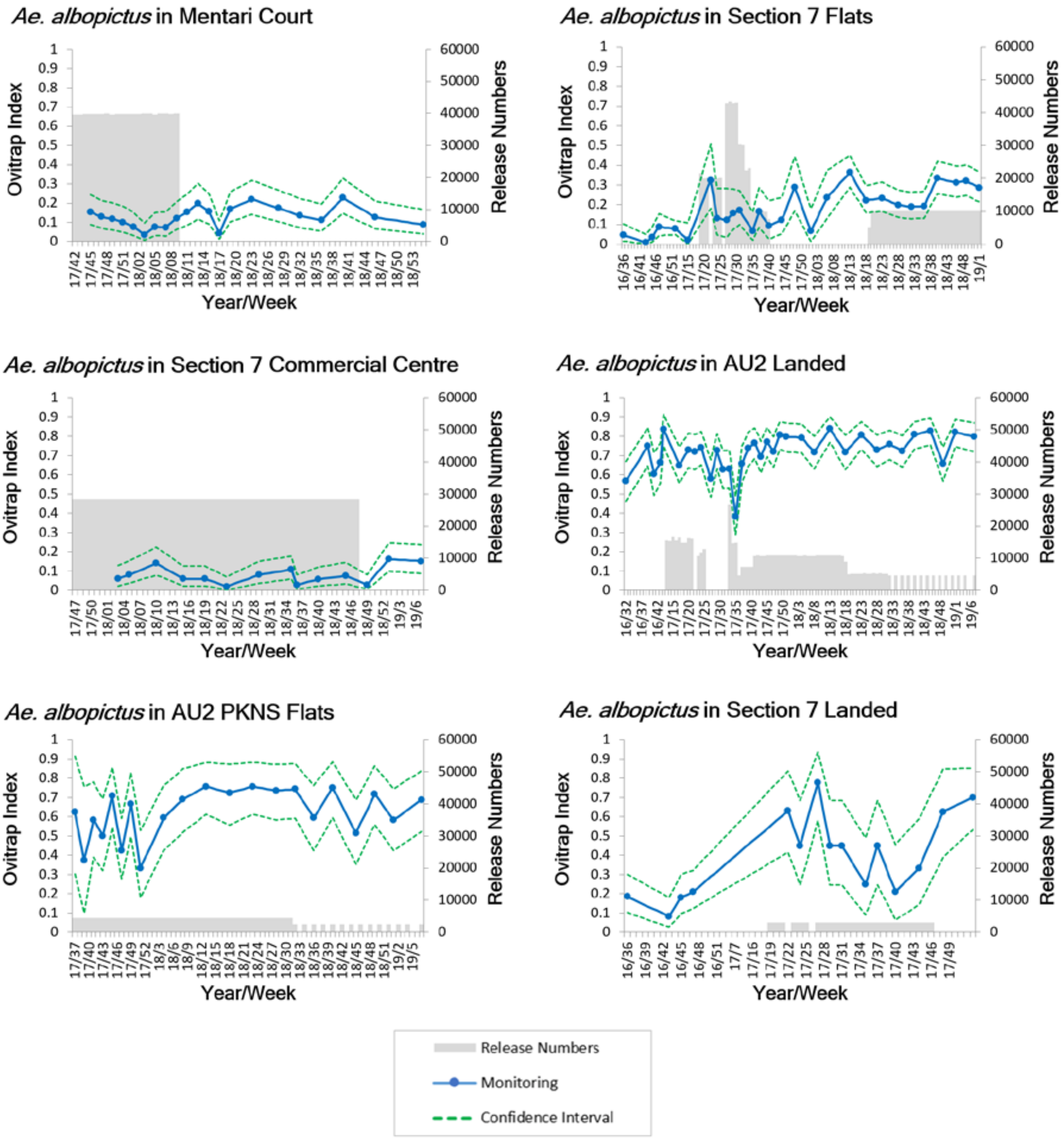

Figure S3. ***Aedes albopictus* population size estimates at release sites, related to figure 2.** Size was measured through an ovitrap index (*Ae. albopictus*-positive traps divided by total number of traps) during the release/monitoring period. Grey shaded areas represent release periods; 95% confidence intervals are shown as dotted lines.

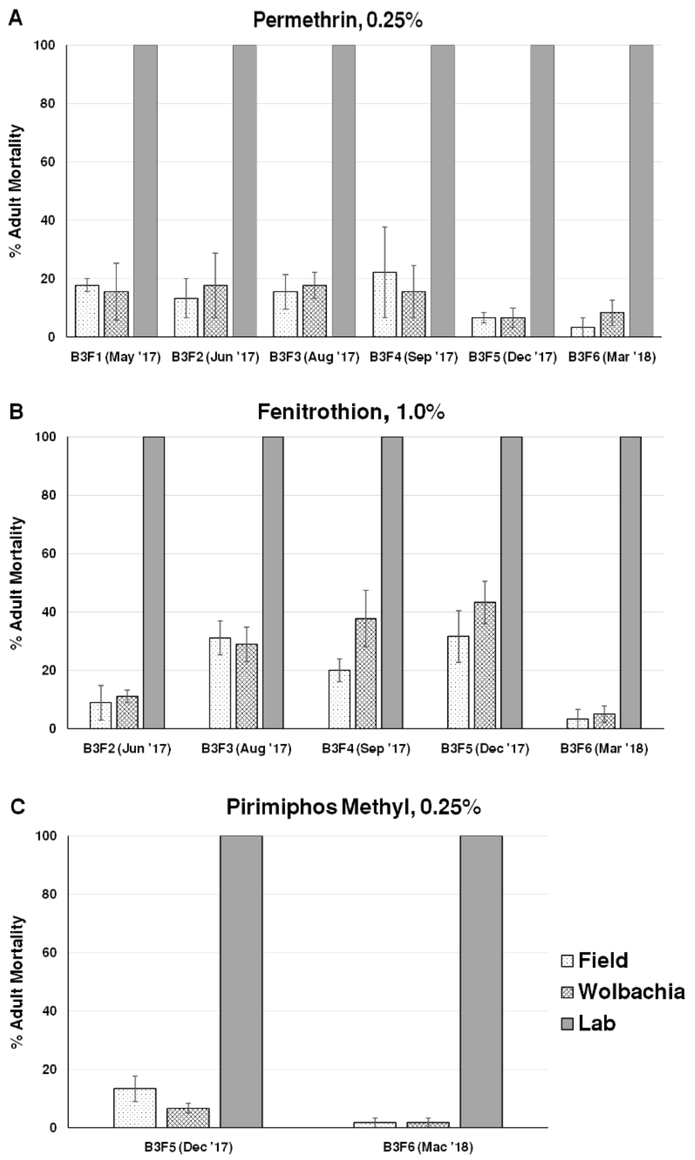

Figure S4. Insecticide susceptibility of the release line ("Wolbachia") compared with the field-derived wild-type population of *Ae. aegypti* ("Field") and a laboratory insecticide susceptible line ("Lab"), related to STAR Methods. Lines were tested against three pesticides: permethrin, fenitrothion, and pirimiphos methyl. Bioassays measured adult mortality at discriminating doses. Error bars represent standard errors.

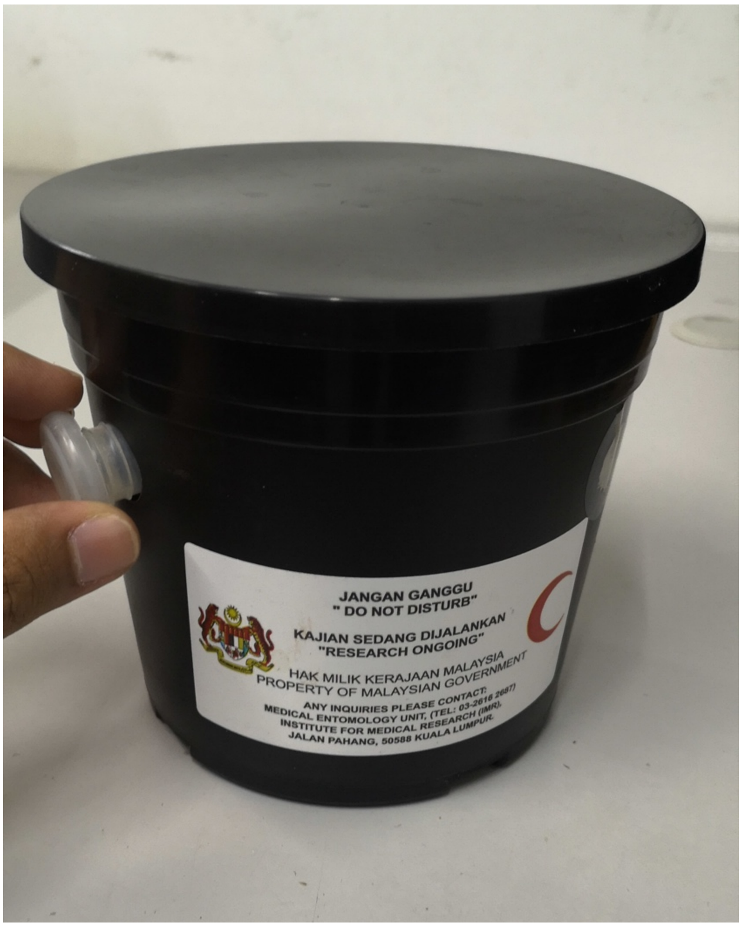

Figure S5. Example of an egg release container as used in the Section 7 Commercial Centre site, related to Figure 3. Note the 2 cm diameter release holes and plug, removed to allow escape of adult mosquitoes.

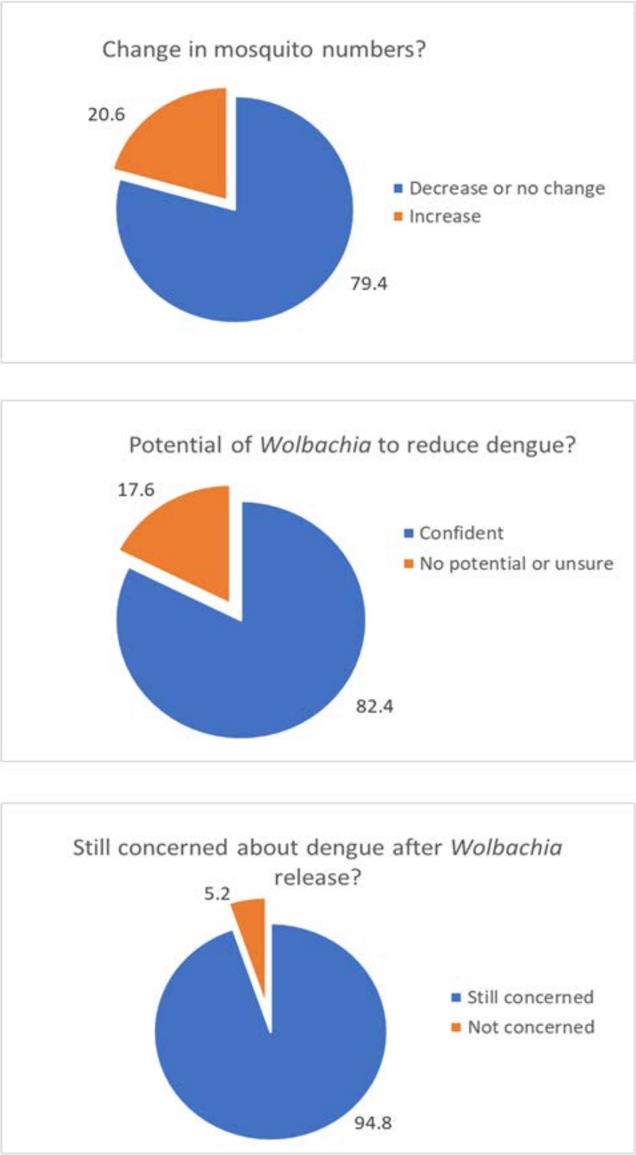

Figure S6. Post-release survey questionnaire results from community engagement activities in Section 7 Flats, related to figures 2 and 3. Data were collected from a door-to-door survey in the Section 7 Flats release area 2 months after the beginning of the releases. 196 respondents were included, consisting of 40% females, with age classes 29-39 (15%), 40-50 (65%) and >60 (10%).

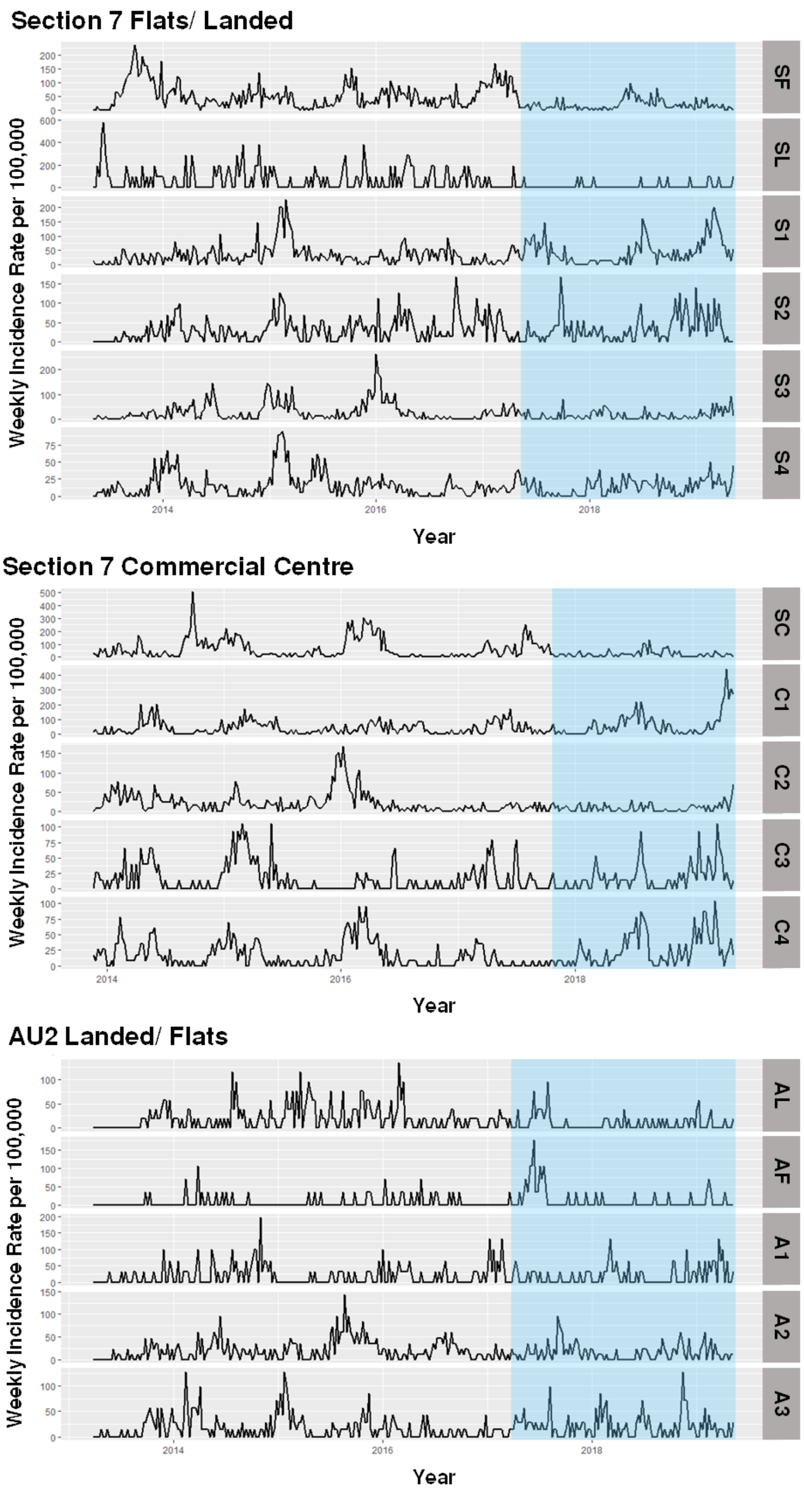

Figure S7. Dengue incidence plots at the Shah Alam Section 7 PKNS flats & Landed, Section 7 Commercial Centre, and AU2 Landed / Flats release and matched control sites, related to Figure 4. SF, SL, SC, AL and AF represents release areas on different sites. See Table 1 for additional details on control sites. The periods during and after commencement of *Wolbachia* releases are indicated by blue regions.
