# Supplementary material for: Establishment of Wolbachia Strain wAlbB in Malaysian Populations of Aedes aegypti for Dengue Control
Source: Curr Biol. 2019 Dec 16;29(24):4241–4248.e5. doi: 10.1016/j.cub.2019.11.007 (PMC6926472; doi:10.1016/j.cub.2019.11.007)
Supplement: Document S2. Article plus Supplemental Information [file mmc2.pdf]

Current Biology

Article

Establishment of *Wolbachia* Strain *w*AlbB in Malaysian Populations of *Aedes aegypti* for Dengue Control

Highlights

- The *w*AlbB strain of *Wolbachia* bacteria was established in *Aedes aegypti* populations
- *Wolbachia* frequencies remained high and stable at some sites
- Dengue incidence decreased at release sites

Authors

Wasi A. Nazni, Ary A. Hoffmann, Ahmad NoorAfizah, ..., Balvinder S. Gill, Han Lim Lee, Steven P. Sinkins

Correspondence

nazni@imr.gov.my (W.A.N.),
ary@unimelb.edu.au (A.A.H.),
steven.sinkins@glasgow.ac.uk (S.P.S.)

In Brief

Nazni et al. show that the *w*AlbB *Wolbachia* infection can successfully invade Malaysian field populations of *Aedes aegypti* mosquitoes, which are responsible for dengue transmission. There was a decrease in dengue incidence at the release sites, highlighting the potential of this strategy to suppress dengue in hot tropical environments.

Nazni et al., 2019, Current Biology *29*, 4241–4248
December 16, 2019 Crown Copyright © 2019 Published by Elsevier Ltd.
https://doi.org/10.1016/j.cub.2019.11.007

Current Biology
Article

Establishment of *Wolbachia* Strain *w*AlbB in Malaysian Populations of *Aedes aegypti* for Dengue Control

Wasi A. Nazni,[1,8,*] Ary A. Hoffmann,[2,8,*] Ahmad NoorAfizah,[1] Yoon Ling Cheong,[1] Maria V. Mancini,[3] Nicholas Golding,[4] Ghazali M.R. Kamarul,[1] Mohd A.K. Arif,[1] Hasim Thohir,[1] Halim NurSyamimi,[1] M. Zabari ZatilAqmar,[1] Mazni NurRuqqayah,[1] Amran NorSyazwani,[1] Azmi Faiz,[1] Francis-Rudin M.N. Irfan,[1] Subramaniam Rubaaini,[1] Nasir Nuradila,[1] Nasir M.N. Nizam,[1] Saidin M. Irwan,[1] Nancy M. Endersby-Harshman,[2] Vanessa L. White,[2] Thomas H. Ant,[3] Christie S. Herd,[3] Asim H. Hasnor,[5] Rahman AbuBakar,[5] Dusa M. Hapsah,[5] Khairuddin Khadijah,[6] Denim Kamilan,[6] Soo Cheng Lee,[6] Yusof M. Paid,[6] Kamaludin Fadzilah,[1] Omar Topek,[7] Balvinder S. Gill,[1] Han Lim Lee,[1] and Steven P. Sinkins[3,9,*]

[1]Institute for Medical Research, Ministry of Health Malaysia, Jalan Pahang 50588, Kuala Lumpur, Malaysia
[2]University of Melbourne, Bio21 Institute and the School of BioSciences, 30 Flemington Road, Parkville, VIC 3052, Australia
[3]MRC-University of Glasgow Centre for Virus Research, 464 Bearsden Road, Glasgow G61 1QH, UK
[4]University of Melbourne, School of BioSciences, Royal Parade, Parkville, VIC 3052, Australia
[5]Institute for Health Behavioural Research (IPTK), Ministry of Health Malaysia, Jln Rumah Sakit Bangsar 50590, Kuala Lumpur, Malaysia
[6]Petaling District Health Office, Ministry of Health Malaysia, SS 6, 47301 Petaling Jaya, Selangor Selangor, Malaysia
[7]Vector Borne Disease Control Section, Disease Control Division, Ministry of Health Malaysia, Complex E, Block E10, Persiaran Sultan Sallahuddin Abdul Aziz Shah, Presint 1, 62000 Putrajaya, Malaysia
[8]These authors contributed equally
[9]Lead Contact
*Correspondence: nazni@imr.gov.my (W.A.N.), ary@unimelb.edu.au (A.A.H.), steven.sinkins@glasgow.ac.uk (S.P.S.)
https://doi.org/10.1016/j.cub.2019.11.007

SUMMARY

Dengue has enormous health impacts globally. A novel approach to decrease dengue incidence involves the introduction of *Wolbachia* endosymbionts that block dengue virus transmission into populations of the primary vector mosquito, *Aedes aegypti*. The *w*Mel *Wolbachia* strain has previously been trialed in open releases of *Ae. aegypti*; however, the *w*AlbB strain has been shown to maintain higher density than *w*Mel at high larval rearing temperatures. Releases of *Ae. aegypti* mosquitoes carrying *w*AlbB were carried out in 6 diverse sites in greater Kuala Lumpur, Malaysia, with high endemic dengue transmission. The strain was successfully established and maintained at very high population frequency at some sites or persisted with additional releases following fluctuations at other sites. Based on passive case monitoring, reduced human dengue incidence was observed in the release sites when compared to control sites. The *w*AlbB strain of *Wolbachia* provides a promising option as a tool for dengue control, particularly in very hot climates.

INTRODUCTION

There are around 90 million symptomatic cases of dengue each year [1], with severe disease in around 1% of cases, including life-threatening hemorrhage or shock syndrome. Reducing abundance of the primary vector mosquito *Aedes aegypti* using insecticides and breeding site reduction remain the main strategies for dengue control but are relatively ineffective. The introduction of *Wolbachia* endosymbionts into *Ae. aegypti* and demonstration of dengue transmission blocking [2–6] have been followed by the use of the *w*Mel strain (from *Drosophila melanogaster*) for "population replacement," resulting in this strain reaching and maintaining a high and stable frequency in north Queensland [7–9], where there has been a near-cessation of imported dengue outbreaks [10].

Wolbachia replacement involves the induction of cytoplasmic incompatibility (CI), a reproductive manipulation imposing a pattern of crossing sterility that provides an advantage to *Wolbachia*-carrying females. CI enables rapid spread to high frequency in insect populations once a threshold frequency has been exceeded, depending on host fitness parameters, and CI and maternal transmission rates [7, 11, 12]. Different *Wolbachia* strains vary considerably in their effects on *Ae. aegypti* fitness parameters [13–16], and thus their population invasion and maintenance capacity (indeed, strain *w*MelPop, a higher-replicating variant of *w*Mel with high fitness cost, was unable to maintain itself in *Ae. aegypti* populations [15]).

Recent reports suggested that *w*Mel may show reduced density and cytoplasmic incompatibility when *Ae. aegypti* larvae are reared at high temperatures [13, 14, 16], which also matched temperatures previously recorded in wild *Ae. aegypti* larval sites elsewhere [17]; larvae in containers exposed to sunlight for part of the day would experience even higher temperatures than the recorded ambient air temperatures. However, *w*AlbB proved to be much less susceptible to the effects of similar high rearing temperatures [14, 16]. This suggests that *w*AlbB might be well suited for population replacement in hot environments, given its ability to effectively block transmission of dengue and other arboviruses [5]; *w*AlbB has not previously been deployed in this way.

Crown Copyright © 2019 Published by Elsevier Ltd.
This is an open access article under the CC BY license (http://creativecommons.org/licenses/by/4.0/).

Table 1. Information on Release and Control Sites Identified from Criteria Discussed in Main Text

Type of Site	Site Name	Blocks (bl), Floors (f)	Dengue Incidence[a]	Approx. No. Households	Area (m^2)
Release-MH	Mentari Court	7bl, 18f	7,371.41	3,469	90,267
Control-M1	Lagoon Perdana	7bl, 18f	4,974.05	3,322	46,713
Control-M2	PJU 8 Apartment Flora Damansara	8bl, 24f	6,937.23	2,200	74,726
Control-M3	Seksyen 13 Apartment Brunsfield Riverview	3bl, 14f	11,725.74	532	10,329
Control-M4	Seksyen 13 Apartment Perdana	4bl, 5–10f	11,977.35	656	28,486
Control-M5	Desa Mentari	11bl, 12–18f	2,566.06	6,560	54,126
Control-M6	Kelana Puteri and Putera Condominium	11bl, 16–20f	7,726.39	1,322	56,586
Control-M7	Section U2 Apartment Ilham	4bl, 18f	9,176.59	576	16,366
Control-M8	Section 7 Apartment Baiduri	4bl, 9–10f	8,547.01	312	21,000
Release-SF	Section 7 Flats	51bl, 5f	11,539.09	2,930	276,261
Release-SL	Section 7 Landed	Landed Houses	14,592.93	248	113,817
Control-S1	Flat Section 6, 7 (Bl52-58), and 8	42bl, 5f	6,850.23	1,790	154,060
Control-S2	Flat Section 16, 17, and 18	68bl, 4–5f	6,341.79	1,712	167,197
Control-S3	Flat Section 19 and 20	37bl, 5f	6,196.28	1,806	170,790
Control-S4	Flat Section 24	77bl, 5f	3,504.36	4,260	151,620
Release-SC	Section 7 Commercial Centre	15bl, 4–5f Shop Apartment	13,325.22	1,408	119,025
Control-C1	Section 15 (Dataran Otomobil)	18bl, 4–5f	9,042.88	1,406	76,048
Control-C2	Flat Section 27	31bl, 4–8f	4,715.82	3,100	157,364
Control-C3	Flat and Apartment Section U3	17bl, 5f	3,677.25	1,800	104,763
Control-C4	Apartment Section U5	23bl, 5–7f	3,681.08	2,736	139,404
Release-AL	AU2 Landed, Keramat	Landed Houses	4,170.03	1,239	727,548
Release-AF	AU2 PKNS Flats	18bl, 4f	1,452.66	672	50,598
Control-A1	AU5 Landed, Keramat	landed houses	4,176.53	724	585,476
Control-A2	Taman Samudera and Taman Sunway, Batu Caves	landed houses	4,270.45	1,996	759,719
Control-A3	Bandar Tasik Puteri Fasa 6 and 7, Rawang	landed houses	3,482.59	1,675	841,977

Release sites are highlighted, followed by matched control sites for those release site(s).
[a]Rate per 100,000, from January 2013 until start of intervention.

The primary aim of these field trials was to assess whether *w*AlbB can be established and maintained at high frequency in urban *Ae. aegypti* in greater Kuala Lumpur, Malaysia, and what conditions are most conducive to establishment. In Malaysia over 100,000 dengue cases were reported in 2016, with an annual cost estimated at $175 million [18, 19]. Extended periods with daily peak temperatures exceeding 36°C occur in Kuala Lumpur. In light of *Wolbachia* strain difference in temperature responses, which may impact both the *Wolbachia* population frequency and the efficacy of dengue blocking in wild populations, this hot tropical environment provides an opportunity to test whether *w*AlbB-carrying *Ae. aegypti* with different effects on host fitness components to *w*Mel [14, 16] can invade an area where dengue is endemic. Information was also collected on dengue incidence in release areas and in matched control sites.

RESULTS

Site Selection

Intervention sites with persistent occurrence of dengue over the previous 4 years were selected (Table 1), in accordance with a WHO-recommended criterion for dengue intervention trial design [20]. Four primary intervention sites were chosen in Selangor State to represent different building types in order to explore *Wolbachia w*AlbB spread and maintenance dynamics in different settings (Table 1; Figure S1): high-rise (18 floor) apartment buildings, 4- to 5-floor flats, 5-floor combined shop and flat buildings, landed terraces, and landed houses (Figure 1). For two of the sites, releases were also conducted at adjacent smaller secondary sites with different building type. Where possible, boundaries to mosquito movement on at least a portion of the perimeter were incorporated in order to minimize immigration from surrounding areas—highways of six lanes and above, rivers, grassland, and park areas [21]. Estimates of *Aedes* species composition and population size over time were obtained using ovitraps (Figures 2, S2, and S3). *Ae. albopictus* is present at all the sites (Figure S3) (the latter is to be targeted subsequently for replacement releases using dengue-blocking strains) [22, 23]. Community engagement is a very important component of *Wolbachia* transmission-blocking programs, given that biting female mosquitoes must be released into the environment; community consent

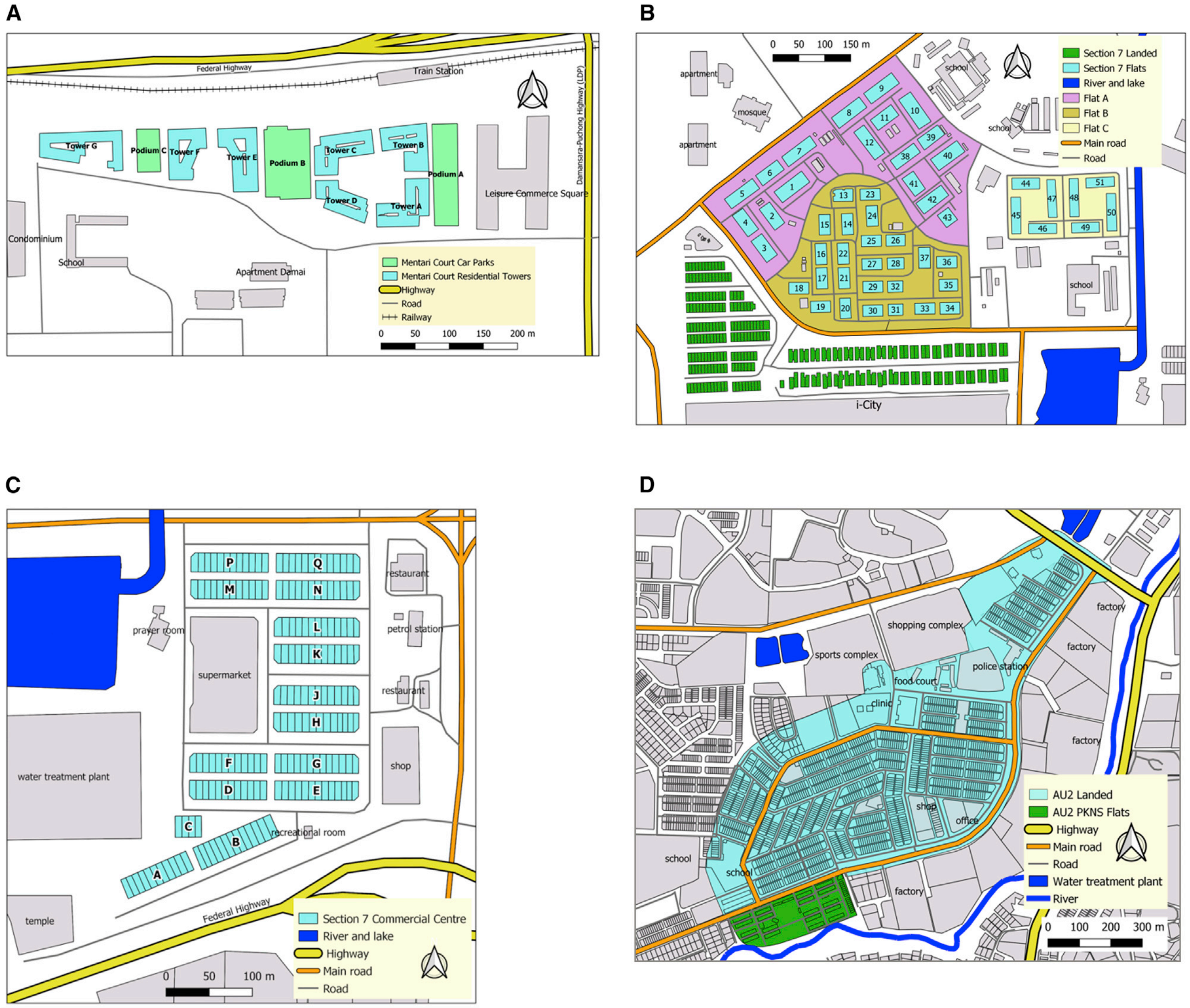

Figure 1. Maps of Six Release Zones

(A) Mentari Court.

(B) Section 7 Flats and Section 7 Landed.

(C) Section 7 Commercial Centre.

(D) AU2 landed and AU2 PKNS flats.

See also Figure S1.

and strong support for releases were obtained in all sites (STAR Methods).

Releases

In order to obtain a fit, locally adapted, and competitive *Ae. aegypti w*AlbB line for release [16], four generations of back-crossing to field-collected local material were carried out. An important factor in relative fitness is insecticide resistance, given increasing levels of resistance [24] (particularly to pyrethroids). Susceptibility was compared between F1 adults from field-collected individuals and the release line using bioassays and found to be similar for pyrethroids, as well as to the organophosphates fenitrothion and pirimiphos (Figure S4). The backcrossed *Ae. aegypti w*AlbB line was mass reared in preparation for releases. Wing measurements taken from mass-reared adults were in the range expected to produce fit, competitive release mosquitoes based on studies with Australian *Ae. aegypti* [25, 26] (average [SD] for males 2.28 [0.10] mm, females 2.96 [0.11] mm).

Wolbachia invasion depends on it exceeding a frequency dictated by fitness effects, incompatibility, and maternal transmission; this was used to estimate required release rate with the ovitrap index (traps positive for *Ae. aegypti* divided by total traps per site) providing an estimate of population size [7]. Adult mosquitoes were released weekly in the morning on a pre-determined grid (with one cup of 50 mosquitoes released on a grid on ground and second floors in Section 7 and on every third floor in Mentari Court). After around 4 weeks of releases, *Wolbachia*

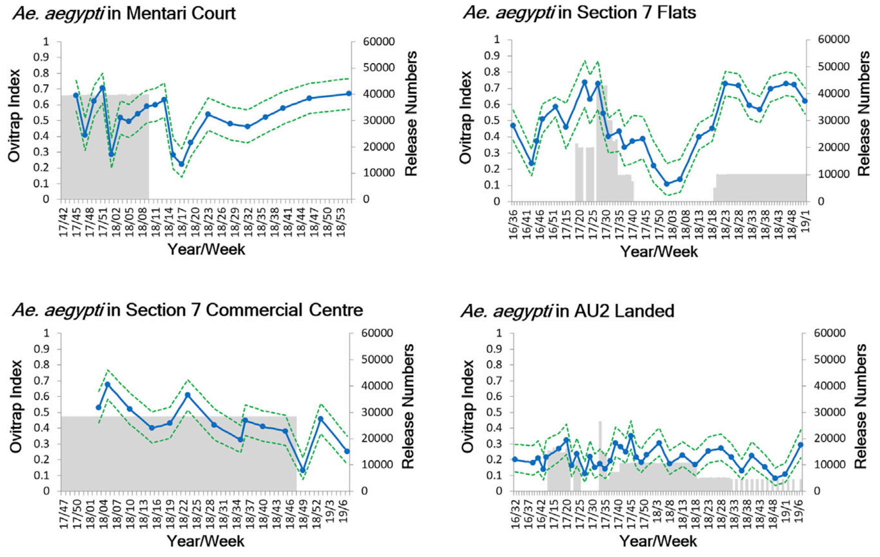

Figure 2. *Ae. aegypti* Population Size Estimates at Release Sites

Ovitrap index (*Ae. aegypti*-positive traps divided by total number of traps) measured during the release and monitoring period. Gray shaded areas represent release periods; 95% confidence intervals are shown as dotted lines. See also Figures S2, S3, and S6.

frequency monitoring commenced using ovitraps, with the resulting eggs returned to the laboratory and raised to adults, and a selection of the *Ae. aegypti* samples from each trap used for *Wolbachia* qPCR analysis [27]. In one of the sites, Section 7 Commercial Centre, eggs rather than adults were released: covered containers to which approximately 200 eggs in 400 mL water with larval food had been added (Figure S5) were left out for 2 weeks for the adults to emerge on site.

Changes in *Wolbachia* and Mosquito Numbers

Wolbachia frequency rose rapidly to over 80% at all sites (Figure 3). Following the cessation of releases, the *Wolbachia* frequency has remained stable and high (98% 12 months after releases ceased) in Mentari Court. At the AU2 and Section 7 Flat sites, the frequency exceeded 95% but subsequently fluctuated following cessation of releases. Immigration of *Wolbachia*-free mosquitoes from surrounding untreated areas can reduce *Wolbachia* frequency where there is a low population size and relatively weak boundary barriers to mosquito population movement

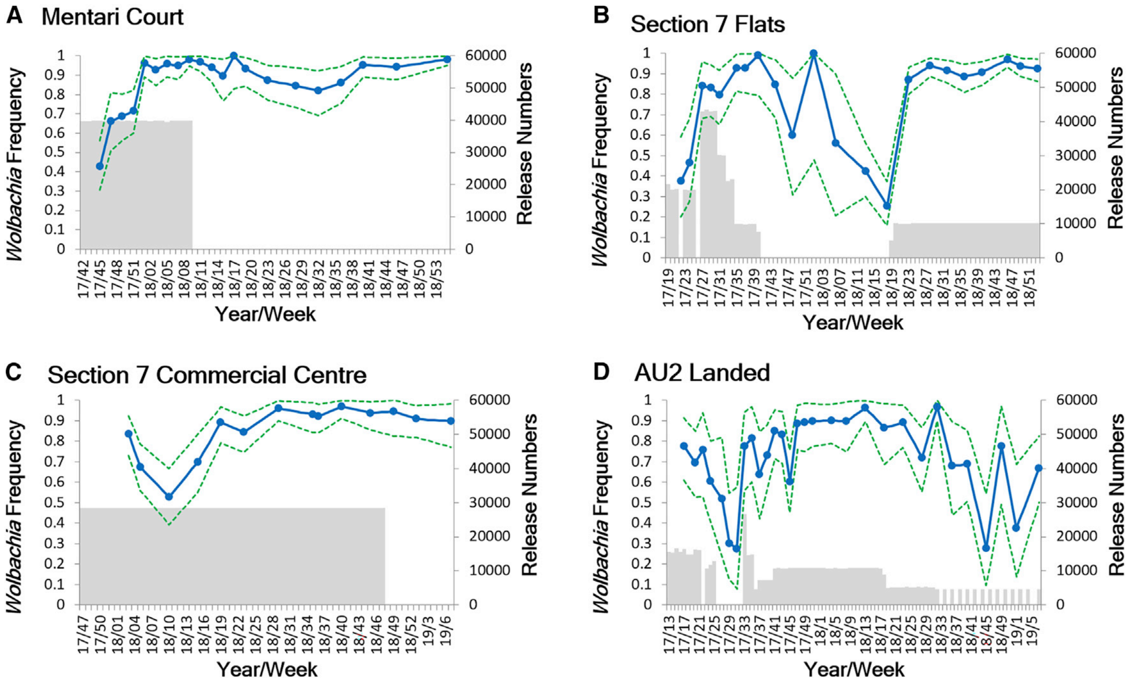

Figure 3. *Wolbachia* Frequency during and after Releases

Monitoring was conducted using ovitrapping and qPCR.

(A) Mentari Court.

(B) Shah Alam Section 7 Flats.

(C) Shah Alam Section 7 Commercial Centre.

(D) AU2 Landed. Release numbers are shown in gray shading; 95% confidence intervals are shown as dotted lines.

See also Figures S2, S5, and S6.

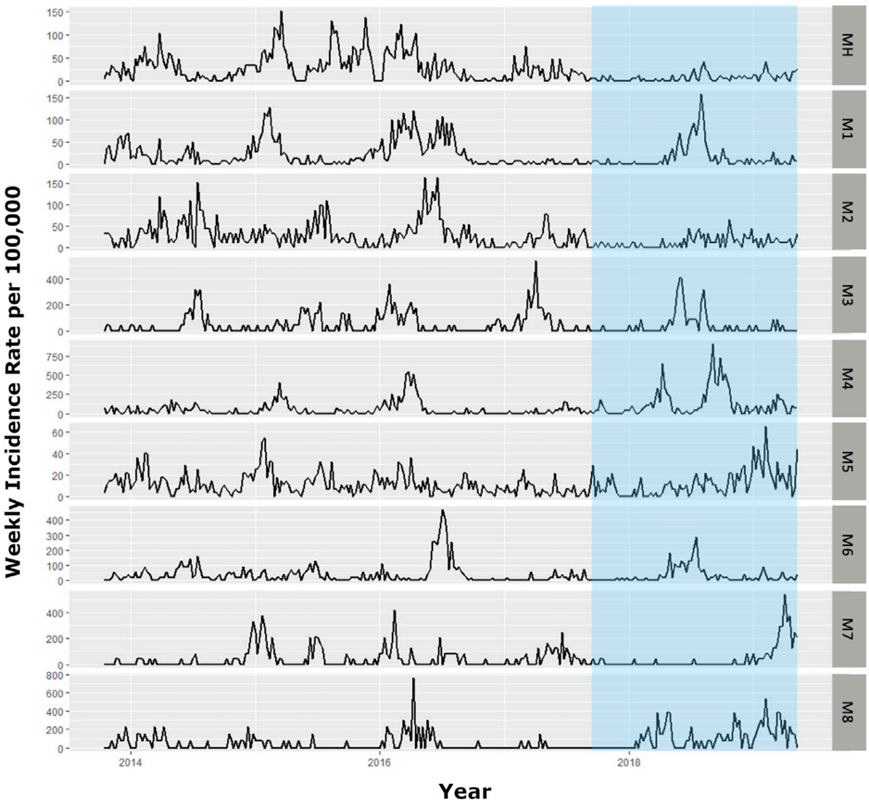

Figure 4. Dengue Incidence from 2013 in Mentari Court (MH) and Matched Control Sites (M 1–8)

The periods during and after commencement of *w*AlbB-carrying *Ae. aegypti* releases are indicated by blue (for other sites and their matched controls, see Figure S7). Incidence is calculated as total confirmed dengue cases per total population * 100,000. See also Figure S7.

[21], which occurred at these locations; also in Section 7 Flats a 29-hectare construction site ("i-City"), 159 m from the release zone, likely provided larval breeding sites and a source of wild-type *Wolbachia*-free migrants. Therefore, it was decided to resume releases at a lower release rate, whereupon *Wolbachia* frequencies rapidly rose again (Figure 3).

Population size monitoring before and during the releases (Figure 2) also confirmed that there were no major population spikes associated with the release phase in the sites; this is expected given that mating between released males and wild females results in embryo death due to cytoplasmic incompatibility, balancing the population-increasing effects of female releases. In fact, in some sites post-*Wolbachia* establishment *Ae. aegypti* population density appeared to be lower than previously recorded based on ovitrap index (Figure 2). More data are needed to support this observation, but the trend is consistent with a population-suppressing effect caused by the fitness cost associated with *w*AlbB, particularly with respect to a steadily increasing mortality over time of desiccated eggs when added to water for hatching [16]. Areas with a higher proportion of temporary rather than permanent breeding sites, where periodic flushing of quiescent eggs is important, are predicted to experience a higher level of population suppression following invasion. Post-release community surveys revealed that most residents did not notice any increase in mosquito biting (Figure S6).

Dengue Incidence

Human dengue incidence from 2013 to 2019 was compared between release sites and matched control sites, based on data recorded by the Malaysian National Dengue Surveillance System. Control sites were selected for comparable dengue incidence to the release sites in the period from 2013 to the start of releases (Table 1), within the same district as the release site where possible, to ensure similar non-*Wolbachia* dengue control activities (except that a wider area was used for some of the Mentari Court sites, in order to ensure that some sites with similar or higher incidence compared to the release site could be identified), and building type of the "primary" site in each location, to meet similar mosquito and human population characteristics (size, movement, etc.) (Table 1). Dengue incidence was reduced following releases in all intervention sites (Figures 4 and S7). A Bayesian time series model [28–30] produced an estimate of dengue case reduction of 40.3% over all intervention sites (95% credible interval 5.06–64.59) (Figure 5), with posterior probability of a reduction in intervention sites post-release of 0.985.

In the 18-floor apartment buildings of Mentari Court, prior to *Wolbachia* releases dengue cases were high since at least 2013 (Figure 4) despite intensive control efforts involving repeated rounds of clean-up "source reduction" coupled with community engagement, and repeated thermal fogging (Figure 6), none of which proved effective in reducing dengue incidence. The introduction of *Wolbachia w*AlbB has, in contrast, reduced dengue cases to a point where insecticide fogging by the local health authorities was no longer considered necessary (Figures 4 and 5).

DISCUSSION

The results indicate the successful introduction of *w*AlbB *Wolbachia* into release sites in Selangor. The frequency of *w*AlbB has remained high at two sites following invasion (Mentari Court and Commercial Centre), and the frequency at Mentari Court is currently still >90%, 2 years after releases were terminated. An important consideration with respect to achieving stable high *Wolbachia* frequency is *Ae. aegypti* population size versus movement; in Mentari Court there is a relatively high population per unit ground area compared to the Section 7 zones, given *Ae. aegypti* were collected in ovitraps all the way up to the 18th floor. This will need to be incorporated into the design of wider strategy

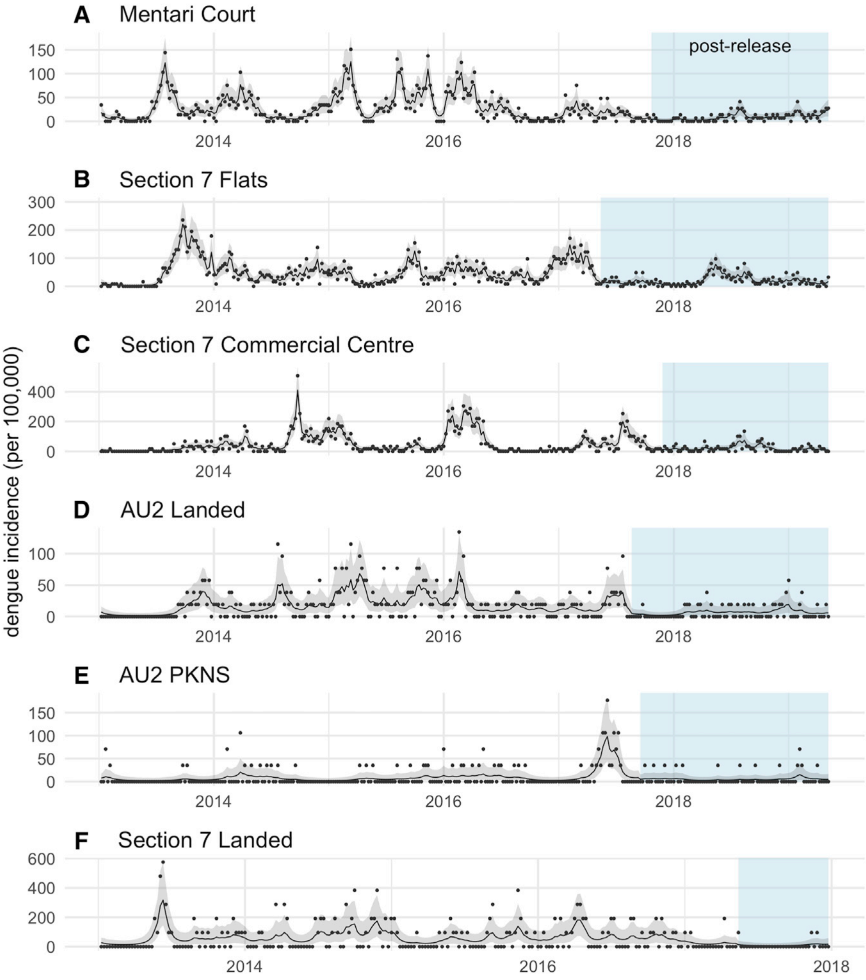

Figure 5. Dengue Reduction following *Wolbachia* Releases

Incidence of confirmed human dengue cases for each week of the study period in the six release sites: (A) Mentari Court, (B) Section 7 Flats, (C) Section 7 Commercial Centre, (D) AU2 Landed, (E) AU2 PKNS, and (F) Section 7 Landed. Black lines and gray shaded areas show the posterior mean and 95% credible intervals of the incidence inferred from a Bayesian time series model. Points represent empirical incidences calculated directly from case data. The periods during and after commencement of *Wolbachia*-carrying *Ae. aegypti* releases are indicated by blue regions.

roll-out; for example, buffer release zones should be incorporated around the perimeter of high-transmission localities that have small area, low *Ae. aegypti* population size, or no clear natural boundaries to *Ae. aegypti* movement. The importance of area size in successful invasions has previously been recognized for *w*Mel introductions in Cairns, Australia, where *Wolbachia* failed to establish at one small site following releases despite successfully establishing at larger nearby release sites [21].

The successful deployment of egg containers for release at the Commercial Centre site has proven to be logistically much less laborious than adult releases and will also greatly facilitate wider roll-out. This reflects the fact that eggs produced in the laboratory can be easily cut into strips and transported to the field, where water and food can be added to containers. In contrast, adult releases require transfer of larvae to release containers where they can pupate and eclose, which requires additional handling of the immature stages by laboratory personnel. Egg containers were previously used in releases of *w*Mel in Townsville, Australia, where they also proved successful [10]. However, if density-dependent processes occur in such containers it could slow the rate of *Wolbachia* invasion [31].

The *w*AlbB strain that has spread to a high prevalence in this study provides an effective blocker of dengue in laboratory studies [16], leading us to investigate a possible impact on dengue in the current study. The dengue incidence data point to an estimated reduction in incidence at release sites of around 40% (with confidence intervals of 5% to 65%) following *Wolbachia* introductions when compared to control sites. Reduction to zero cases would not be an expected outcome even if the strategy is 100% efficient. This is because *Ae. aegypti* bite during the daytime and thus dengue can be acquired outside of the place of residence (for example, at work or school) during the day, and also because *Ae. albopictus* is abundant at these sites (Figure S3) and has yet to be targeted using *Wolbachia* replacement. Both factors make the effect of the intervention more difficult to detect with passive surveillance as was used here; only the effects of *Wolbachia* on local transmission of dengue by *Ae. aegypti* in the release zone are being detected in these comparisons. Nevertheless, clear differences in incidence between intervention and control sites were observed. The overall effect size detected here is thus a conservative estimate. Active surveillance using seroconversion of naive pre-school children would provide a more accurate measure of the effect of the releases on local transmission, given a much higher proportion of locally acquired cases are expected in this cohort [20], but this was beyond the budget and scope of the current study.

In summary, the results clearly demonstrate the capacity of *Wolbachia* strain *w*AlbB in urban *Ae. aegypti* to become established and maintain itself at high frequency in *Ae. aegypti* populations. Releases in a larger number of diverse intervention sites are now being undertaken in conjunction with the Malaysian Ministry of Health to comprehensively evaluate *w*AlbB effects on dengue transmission in different settings. The cessation of fogging in the release zones due to reduced dengue cases also points to the economic sustainability of the approach, given that large sums are spent annually on insecticides for dengue control [19]. Longer periods of dengue monitoring post-release will further increase the accuracy of the estimated effect size of the intervention on dengue incidence, and as more areas become *Wolbachia*-positive the proportion of imported cases will also fall. The

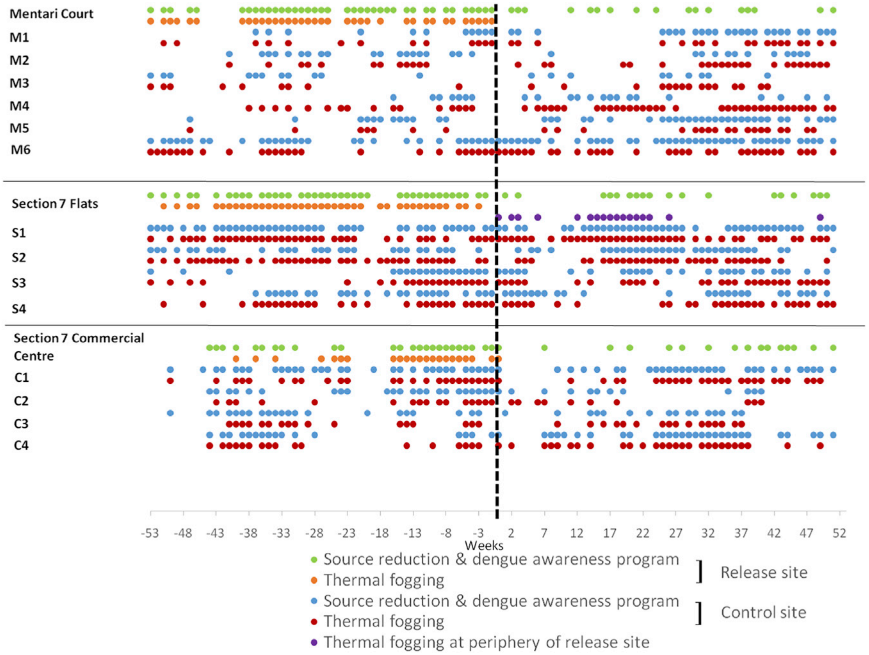

Figure 6. Dengue Control Activities (Other Than *Wolbachia* Release) at the Section 7 Commercial Centre, Mentari Court, and Section 7 PKNS Flats in Release and Matched Control Sites

The date of first *Wolbachia* releases is indicated by the black dashed line, and activities carried out in the year before and year after this date are shown. Data were only available for three release sites. Thermal fogging at the periphery refers to fogging done at nearby construction sites.

establishment of *w*AlbB under high-temperature conditions as reported here points to it being a promising option for deployment in very hot tropical climates. Transmission blocking for other viruses including Zika, chikungunya, and yellow fever could also be achieved using *Wolbachia* [4, 32, 33].

STAR★METHODS

Detailed methods are provided in the online version of this paper and include the following:

- KEY RESOURCES TABLE
- LEAD CONTACT AND MATERIALS AVAILABILITY
- EXPERIMENTAL MODEL AND SUBJECT DETAILS
- METHOD DETAILS
  - Mass rearing
  - Insecticide susceptibility assays
  - Community engagement
  - Mosquito releases
  - Monitoring mosquito population size and *Wolbachia* frequency
  - Dengue control activities
  - Dengue incidence
- QUANTIFICATION AND STATISTICAL ANALYSIS
- DATA AND CODE AVAILABILITY

SUPPLEMENTAL INFORMATION

Supplemental Information can be found online at https://doi.org/10.1016/j.cub.2019.11.007.

ACKNOWLEDGMENTS

The primary funding for the study was provided by Wellcome Trust Award 108508, with additional funding from Wellcome Trust Award 202888 and National Health and Medical Research Council of Australia Awards 1118640 and 1132412 and funding from Ministry of Health Malaysia. The authors thank the Director General of Health, Malaysia, for permission to publish. Thanks are also due to the Deputy Director General of Health (Research & Technical Support), Malaysia, and Director, Institute for Medical Research, Kuala Lumpur, for the support. We acknowledge Datuk Dr. Shahnaz Murad and Dr. Rose Nani Mudin for the support throughout the project.

AUTHOR CONTRIBUTIONS

S.P.S. and A.A.H. conceived the study; W.A.N., A.N.A., Y.L.C., M.V.M., G.M.R.K., M.A.K.A., H.T., H.N.S., M.Z.Z.A., M.N.R., A.N.S., A.F., F.-R.M.N.I., S.R., N.N., N.M.N.N., S.M.I., N.M.E.-H., V.L.W., T.H.A., C.S.H., A.H.H., R.A.B., D.M.H., K.K., D.K., S.C.L., Y.M.P., K.F., and O.T. performed the experimental work; Y.M.P. and K.F. provided resources; O.T. provided cleaned extracted data; A.A.H., Y.L.C., N.G., B.S.G., M.V.M., and S.P.S. analyzed the data; A.A.H., S.P.S., W.A.N., and H.L.L. supervised the project; S.P.S. and A.A.H. wrote the manuscript; and all authors reviewed and edited the manuscript.

DECLARATION OF INTERESTS

The authors declare no competing interests.

Received: September 30, 2019
Revised: October 30, 2019
Accepted: November 1, 2019
Published: November 21, 2019

REFERENCES

1. Bhatt, S., Gething, P.W., Brady, O.J., Messina, J.P., Farlow, A.W., Moyes, C.L., Drake, J.M., Brownstein, J.S., Hoen, A.G., Sankoh, O., et al. (2013). The global distribution and burden of dengue. Nature *496*, 504–507.
2. Xi, Z., Khoo, C.C.H., and Dobson, S.L. (2005). *Wolbachia* establishment and invasion in an *Aedes aegypti* laboratory population. Science *310*, 326–328.

3. McMeniman, C.J., Lane, R.V., Cass, B.N., Fong, A.W., Sidhu, M., Wang, Y.F., and O'Neill, S.L. (2009). Stable introduction of a life-shortening *Wolbachia* infection into the mosquito *Aedes aegypti*. Science *323*, 141–144.
4. Moreira, L.A., Iturbe-Ormaetxe, I., Jeffery, J.A., Lu, G., Pyke, A.T., Hedges, L.M., Rocha, B.C., Hall-Mendelin, S., Day, A., Riegler, M., et al. (2009). A *Wolbachia* symbiont in *Aedes aegypti* limits infection with dengue, Chikungunya, and *Plasmodium*. Cell *139*, 1268–1278.
5. Bian, G., Xu, Y., Lu, P., Xie, Y., and Xi, Z. (2010). The endosymbiotic bacterium *Wolbachia* induces resistance to dengue virus in *Aedes aegypti*. PLoS Pathog. *6*, e1000833.
6. Walker, T., Johnson, P.H., Moreira, L.A., Iturbe-Ormaetxe, I., Frentiu, F.D., McMeniman, C.J., Leong, Y.S., Dong, Y., Axford, J., Kriesner, P., et al. (2011). The *w*Mel *Wolbachia* strain blocks dengue and invades caged *Aedes aegypti* populations. Nature *476*, 450–453.
7. Hoffmann, A.A., Montgomery, B.L., Popovici, J., Iturbe-Ormaetxe, I., Johnson, P.H., Muzzi, F., Greenfield, M., Durkan, M., Leong, Y.S., Dong, Y., et al. (2011). Successful establishment of *Wolbachia* in *Aedes* populations to suppress dengue transmission. Nature *476*, 454–457.
8. Hoffmann, A.A., Iturbe-Ormaetxe, I., Callahan, A.G., Phillips, B.L., Billington, K., Axford, J.K., Montgomery, B., Turley, A.P., and O'Neill, S.L. (2014). Stability of the *w*Mel *Wolbachia* infection following invasion into *Aedes aegypti* populations. PLoS Negl. Trop. Dis. *8*, e3115.
9. Frentiu, F.D., Zakir, T., Walker, T., Popovici, J., Pyke, A.T., van den Hurk, A., McGraw, E.A., and O'Neill, S.L. (2014). Limited dengue virus replication in field-collected *Aedes aegypti* mosquitoes infected with *Wolbachia*. PLoS Negl. Trop. Dis. *8*, e2688.
10. O'Neill, S.L., Ryan, P.A., Turley, A.P., Wilson, G., Retzki, K., Iturbe-Ormaetxe, I., Dong, Y., Kenny, N., Paton, C.J., Ritchie, S.A., et al. (2019). Scaled deployment of *Wolbachia* to protect the community from dengue and other *Aedes* transmitted arboviruses. Gates Open Res *2*, 36.
11. Hancock, P.A., Sinkins, S.P., and Godfray, H.C.J. (2011). Population dynamic models of the spread of *Wolbachia*. Am. Nat. *177*, 323–333.
12. Barton, N.H., and Turelli, M. (2011). Spatial waves of advance with bistable dynamics: cytoplasmic and genetic analogues of Allee effects. Am. Nat. *178*, E48–E75.
13. Ulrich, J.N., Beier, J.C., Devine, G.J., and Hugo, L.E. (2016). Heat sensitivity of *w*Mel *Wolbachia* during *Aedes aegypti* development. PLoS Negl. Trop. Dis. *10*, e0004873.
14. Ross, P.A., Wiwatanaratanabutr, I., Axford, J.K., White, V.L., Endersby-Harshman, N.M., and Hoffmann, A.A. (2017). *Wolbachia* infections in *Aedes aegypti* differ markedly in their response to cyclical heat stress. PLoS Pathog. *13*, e1006006.
15. Nguyen, T.H., Nguyen, H.L., Nguyen, T.Y., Vu, S.N., Tran, N.D., Le, T.N., Vien, Q.M., Bui, T.C., Le, H.T., Kutcher, S., et al. (2015). Field evaluation of the establishment potential of *w*MelPop *Wolbachia* in Australia and Vietnam for dengue control. Parasit. Vectors *8*, 563.
16. Ant, T.H., Herd, C.S., Geoghegan, V., Hoffmann, A.A., and Sinkins, S.P. (2018). The *Wolbachia* strain *w*Au provides highly efficient virus transmission blocking in *Aedes aegypti*. PLoS Pathog. *14*, e1006815.
17. Hemme, R.R., Tank, J.L., Chadee, D.D., and Severson, D.W. (2009). Environmental conditions in water storage drums and influences on *Aedes aegypti* in Trinidad, West Indies. Acta Trop. *112*, 59–66.
18. Crisis Preparedness and Response Centre (2016). Bahagian Kawalan Penyakit, Kementerian Kesihatan Malaysia. http://idengue.arsm.gov.my/.
19. Packierisamy, P.R., Ng, C.W., Dahlui, M., Inbaraj, J., Balan, V.K., Halasa, Y.A., and Shepard, D.S. (2015). Cost of dengue vector control activities in Malaysia. Am. J. Trop. Med. Hyg. *93*, 1020–1027.
20. World Health Organization (2017). How to design vector control efficacy trials. Guidance on phase III vector control field trial design. https://www.who.int/neglected_diseases/vector_ecology/resources/WHO_HTM_NTD_VEM_2017.03/.
21. Schmidt, T.L., Barton, N.H., Rašić, G., Turley, A.P., Montgomery, B.L., Iturbe-Ormaetxe, I., Cook, P.E., Ryan, P.A., Ritchie, S.A., Hoffmann, A.A., et al. (2017). Local introduction and heterogeneous spatial spread of dengue-suppressing *Wolbachia* through an urban population of *Aedes aegypti*. PLoS Biol. *15*, e2001894.
22. Blagrove, M.S.C., Arias-Goeta, C., Di Genua, C., Failloux, A.B., and Sinkins, S.P. (2013). A *Wolbachia w*Mel transinfection in *Aedes albopictus* is not detrimental to host fitness and inhibits Chikungunya virus. PLoS Negl. Trop. Dis. *7*, e2152.
23. Blagrove, M.S.C., Arias-Goeta, C., Failloux, A.B., and Sinkins, S.P. (2012). *Wolbachia* strain *w*Mel induces cytoplasmic incompatibility and blocks dengue transmission in *Aedes albopictus*. Proc. Natl. Acad. Sci. USA *109*, 255–260.
24. Moyes, C.L., Vontas, J., Martins, A.J., Ng, L.C., Koou, S.Y., Dusfour, I., Raghavendra, K., Pinto, J., Corbel, V., David, J.P., and Weetman, D. (2017). Contemporary status of insecticide resistance in the major *Aedes* vectors of arboviruses infecting humans. PLoS Negl. Trop. Dis. *11*, e0005625.
25. Ross, P.A., Endersby, N.M., Yeap, H.L., and Hoffmann, A.A. (2014). Larval competition extends developmental time and decreases adult size of *w*MelPop *Wolbachia*-infected *Aedes aegypti*. Am. J. Trop. Med. Hyg. *91*, 198–205.
26. Yeap, H.L., Endersby, N.M., Johnson, P.H., Ritchie, S.A., and Hoffmann, A.A. (2013). Body size and wing shape measurements as quality indicators of *Aedes aegypti* mosquitoes destined for field release. Am. J. Trop. Med. Hyg. *89*, 78–92.
27. Lee, S.F., White, V.L., Weeks, A.R., Hoffmann, A.A., and Endersby, N.M. (2012). High-throughput PCR assays to monitor *Wolbachia* infection in the dengue mosquito (*Aedes aegypti*) and *Drosophila simulans*. Appl. Environ. Microbiol. *78*, 4740–4743.
28. Allard, R. (1998). Use of time-series analysis in infectious disease surveillance. Bull. World Health Organ. *76*, 327–333.
29. Golding, N. (2018). R package version 0.3.0.9002. Greta simple scalable statistical modelling in R. https://github.com/greta-dev/greta.
30. Plummer, M., Best, N., Cowles, K., and Vines, K. (2006). CODA: convergence diagnosis and output analysis for MCMC. R News *6*, 7–11.
31. Hancock, P.A., White, V.L., Callahan, A.G., Godfray, C.H.J., Hoffmann, A.A., and Ritchie, S.A. (2016). Density-dependent population dynamics in *Aedes aegypti* slow the spread of *w*Mel *Wolbachia*. J. Appl. Ecol. *53*, 785–793.
32. van den Hurk, A.F., Hall-Mendelin, S., Pyke, A.T., Frentiu, F.D., McElroy, K., Day, A., Higgs, S., and O'Neill, S.L. (2012). Impact of *Wolbachia* on infection with chikungunya and yellow fever viruses in the mosquito vector *Aedes aegypti*. PLoS Negl. Trop. Dis. *6*, e1892.
33. Dutra, H.L.C., Rocha, M.N., Dias, F.B.S., Mansur, S.B., Caragata, E.P., and Moreira, L.A. (2016). *Wolbachia* blocks currently circulating Zika virus isolates in Brazilian *Aedes aegypti* mosquitoes. Cell Host Microbe *19*, 771–774.

STAR★METHODS

KEY RESOURCES TABLE

REAGENT or RESOURCE	SOURCE	IDENTIFIER
Biological Samples		
wAlbB carrying *Aedes aegypti*	Microinjected line from Ant et al. [16]	N/A
Chemicals, Peptides, and Recombinant Proteins		
Fenithrothion	Supplies for Insecticide Resistance Monitoring, WHO Collaborating Centre, Vector Control Research Unit, University Science Malaysia Penang; https://www.usm.my	N/A
Permethrin	Supplies for Insecticide Resistance Monitoring, WHO Collaborating Centre, Vector Control Research Unit, University Science Malaysia Penang; https://www.usm.my	N/A
Pirimiphos methyl	Supplies for Insecticide Resistance Monitoring, WHO Collaborating Centre, Vector Control Research Unit, University Science Malaysia Penang; https://www.usm.my	N/A
Deposited Data		
Data generated during the study	This study	https://doi.org/10.17632/v8vn35zj3g.1
Software and Algorithms		
Analysis code	This study	https://doi.org/10.5281/zenodo.3520216
R package vegan	N/A	https://cran.r-project.org/web/packages/vegan/vegan.pdf
DIMAS Professional version 5.0	LESO Instruments (M) Sdn Bhd Malaysia	https://www.machinetools.com
greta	N/A	https://github.com/greta-dev/greta
coda	N/A	https://rdrr.io/cran/coda/

LEAD CONTACT AND MATERIALS AVAILABILITY

Further information and requests for resources should be directed to and will be fulfilled by the lead contact, Steven Sinkins (steven.sinkins@glasgow.ac.uk). This study did not generate new unique reagents. There are MTA restrictions to the availability of the *Ae. aegypti*-*w*AlbB line, due to collaborative agreements put in place to ensure all further releases of this line are conducted in a carefully controlled manner.

EXPERIMENTAL MODEL AND SUBJECT DETAILS

The *w*AlbB *Wolbachia* line that formed the basis for the releases had previously been produced by microinjection [16] into an uninfected *Aedes aegypti* line originating from Kuala Lumpur. The line was transferred from Glasgow University to the Institute for Medical Research, Kuala Lumpur, where backcrosses were undertaken prior to mass rearing to maximize the fitness and competitiveness of the mosquitoes to be released.

Backcrossing to freshly field-collected material was carried out. A total of 100 3-5 day-old females from the *w*AlbB line were placed into 24 × 24 × 24 cm cages, together with the same number of 3-5 day-old males per cage of F1 / F2 mosquitoes from Shah Alam. After being left for mating for 2-3 days, they were allowed to blood feed on mice (Malaysian National Institute of Health approval number NMRR-16-297-28898). F1 females were then crossed to males of the field strain, again F1 / F2, to obtain the backcross one (B1) generation. The process was repeated twice to obtain generation B3. The presence of *Wolbachia* at 100% frequency was confirmed in the B3 generation by qPCR as described below.

Prior to releases being initiated, a risk assessment was completed and permission was obtained for *Wolbachia* releases after being examined by NIH and unconditionally approved by the Medical Research and Ethics Committee (MREC), Ministry of Health Malaysia (reference number KKM/NIHSEC/P16-566). An intensive program of community engagement was undertaken at each site as detailed below. Mosquito releases were undertaken in 6 sites in the Petaling and Gombak Districts of Selangor (Table 1; Figure S1) where matched control sites were also identified. Detailed maps of the release sites are provided in Figure 1.

Wolbachia frequencies in release sites were monitored with mosquito samples obtained from ovitraps. To obtain an indication of changes in mosquito population size, numbers of both the target mosquito species (*Aedes aegypti*) and another common mosquito species (*Aedes albopictus*) were also monitored with the ovitraps. In addition to being used in release sites, ovitraps were used in several control sites (Table 1) to monitor changes in mosquito abundance.

Notified dengue cases in all release and control sites were recorded from local clinics and hospitals. These cases had been serologically confirmed and form part of the Malaysian National Dengue Surveillance System. Impacts of releases on dengue impacts were assessed through Bayesian models which compared dengue incidence before and after releases were initiated in release and control sites.

METHOD DETAILS

Mass rearing

To mass rear mosquitoes for release, eggs were weighed on paper in 0.225 g lots (approx. 15000 eggs) and submerged in tap water (after 1 week of desiccation) in plastic containers (16 × 16 × 8 cm), after being exposed to an air vacuum to stimulate hatching. Eggs were then transferred to a 36 × 26 × 5.5 cm trays containing 1 L of tap water. Two days after hatching, L2 larvae from the 15000 eggs were sieved and transferred into 500 mL beakers filled with seasoned water (tap water stored overnight to dechlorinate). Aliquots of larvae were taken from the beaker using 10 mL plastic pipette tips (50 larvae per aliquoted mL). Ten aliquots (1 mL each) were placed into 36 × 26 × 5.5 cm trays filled with 1 L seasoned water. Cow liver powder (Difco, Becton, Dickinson and Co, Sparks, MD USA) and half-cooked cow liver were supplied to larvae daily at rates of 0.06, 0.08, 0.16, 0.31, 0.64, 0.32, 0.32, 0.32, 0.16, 0.08 and 0.06 mg per larva from days 1 to 11.

Pupae were transferred into small plastic containers with seasoned water and transferred into a mosquito cage (approximately 1000 pupae per cage). The mosquitoes were supplied with 10% sucrose solution with vitamin B complex. For blood feeding, two laboratory-reared mice were left in the cage overnight. Two days post-feeding, ovitraps (diameter 7 cm, height 9 cm) lined with A4 paper and with 200 mL of seasoned water were left for egg laying for four days. The paper was dried and then sealed in a plastic bag until use.

Wing measurements were carried out periodically on 15 mass reared males and 15 females for quality control. Both left and right wings of individual mosquitoes were dissected and measured under saline using DIMAS Version 5.0 Professional Edition and an SSZ-T-3.5x mm graticule.

Insecticide susceptibility assays

Adult bioassays for insecticide resistance were carried out using strains from field *Ae. aegypti* from Shah Alam and *Wolbachia*-carrying *Ae. aegypti*, together with a susceptible Kuala Lumpur laboratory strain that has been in culture for over 1000 generations. One commonly-used pyrethroid (permethrin) and two organophosphates (fenithrothion and pirimiphos methyl) were tested. For the field-derived samples, 20-100 larvae were sourced from ovitraps and reared for 1-5 generations in the lab. The *Wolbachia* mosquito colony was also derived from Shah Alam (see above). Larvae were raised on liver powder and 3-5 day-old adult females were tested.

The bioassays followed the WHO resistance test (WHO, 2016) using insecticide impregnated papers (Vector Control Research Unit, Universiti Sains Malaysia, Penang. 10-20 adult females were used for each of the three biological replicates. The adults were exposed to papers impregnated with permethrin 0.25%, pirimiphos methyl 0.25% and fenitrothion 1.0%. Two controls were also set up for each treatment. Knockdown was scored every 5 min during a 1 h exposure period. After exposure, the mosquitoes were transferred into paper cups with cotton soaked in 10% sugar solution. Mortality was recorded after 24 h. Tests were repeated during the period that the colony was used for releases, except in the case of pirimiphos methyl that was tested twice. See Figure S4.

Community engagement

Acceptance and support of the community are essential for the releases. Printed educational materials containing clear information (e.g., leaflets, posters, buntings and banners) on *Wolbachia* were displayed and distributed. A website was established for the *Wolbachia* Malaysia Project linked at https://www.imr.gov.my/wolbachia/. The target group to engage was the head of household and communities. To engage the public, interactions were initiated with local government / political / religious and community leaders, using information kits, workshops / roadshows, meetings / briefings, carnivals and home visits. A total of 40 stakeholders and community engagement activities were conducted in places of worship and community halls. Communities and Ahli Dewan Undangan Negeri (State Assemblyman) were also invited to the Institute for Medical Research to experience first-hand the science of *Wolbachia*. These activities were designed so that the communities would possess a sense of belonging to the program, and feel that their involvement was recognized. Public meetings reinforced the ground-level support for the trials as reflected by a willingness of community members to participate in the program. This high level of support continued throughout the release periods.

Engagement by a team from IPTK (Institute for Health Behavioral Research) in all 6 release sites was conducted by undertaking lectures / talks, using brochures, undertaking advertised discussions with resident groups, posting banners & bunting, providing information on a website, as well as by sending messages to resident through WhatsApp, and SMS. There were between 4 and 17 activities at a release site including meetings, briefings, dialog, carnivals and home visits (fewer activities were carried out at sites that were used later in releases as information about the releases spread). All activities involved local political leaders, community leaders and residents. The aim was to get the community's trust in *Wolbachia* releases and the target of the community engagement

team was at least 80% of the population having been exposed to the *Wolbachia* program through one or more of these approaches. The majority of the resident populations in three sites were tenants (65%–70% Flat PKNS Section 7, 60%–70% Mentari Court, and 80% in Section 7 Commercial Centre); tenants were in general less responsive and less involved in lecture activities and group discussion compared to homeowners. In the AU2 Landed, Section 7 Landed and AU2 Flats sites, none of the residents were tenants.

The IPTK team carrying out engagement continuously sought community feedback throughout the period and found that the 80% exposure target was not reached in some release sites. Hence the IPTK team, after discussing with the head of the blocks and apartments as well as Joint Management Board (JMB), decided at these release sites to obtain agreement for project implementation from community leaders and each community Joint Management Board.

In Shah Alam Section 7 Flats and Section 7 Landed sites, the vast majority of residents surveyed agreed to be involved in the *Wolbachia* mosquito release project: 99.6% (650 of 652) of responding households gave their approval for the project. In AU2 Landed and AU2 Flats, 98.4% (501 of 509) of responding households gave their approval to the project. In Shah Alam, of those responding to the survey 62.9% were male and 27.5% female, while in AU2 Keramat, 50.7% were male and 33.2% female; those responding by WhatsApp and URL were unidentified and accounted for 9.7% and 16.1% from Shah Alam and AU2, respectively. In Mentari Court, the target group involved 20 community leaders rather than residents because attendance at resident meetings was low. All community leaders agreed to the release of *Wolbachia* in all the blocks and car parks in Mentari Court. The community engagement in Section 7 Commercial Centre involved briefing community leaders on the *Wolbachia* project by the IPTK team. After engagement, all 14 community leaders agreed for Commercial Centre Section 7 to be included in the project. The community leaders requested *Wolbachia* videos and health promotion materials for distribution to businesses, shops, houses and other premises, and this was provided.

For all release sites, the *Wolbachia* mosquito release progress was shared with the communities by inviting community representatives to the Institute for Medical Research on several occasions. An engagement activity in the community was also run where the development of *Wolbachia* carrying *Aedes aegypti* larvae were monitored in egg release breeding containers directly exposed to sunlight, partially shaded areas and shaded areas.

Feedback surveys on *Wolbachia* interventions were conducted prior to and after releases. Before releases, the public was informed of the program through information kits, workshops / roadshows, meetings / briefings, carnivals and home visits and feedback was obtained on these activities. This pre-release program helped in gaining consent of the community for the releases. After releases, a questionnaire was used to gauge the response and opinion of the community. Questions included the public perceptions to *Wolbachia* releases and dengue, the perception of the mosquito population size before, during and after the releases, the level of confidence in *Wolbachia* reducing dengue cases, opinions on breeding sites reduction after *Wolbachia* releases and level of concern about dengue transmission. Approximately a year after release initiation, the communities from the release sites were again provided with briefings and updates on the progress of the program on four occasions.

Mosquito releases

Releases were carried out in 6 sites (Table 1). The dates for releases in Section 7 Flats, Section 7 Landed, Mentari Court, and Commercial Centre were 13 May 2017, 13 May 2017, 16 October 2017 and 20 November 2017 respectively, while the dates for AU2 Landed and AU2 Flats were 28 March 2017 and 13 September 2017 respectively. The 1st monitoring was conducted after 4 weeks of adult releases. However, for the egg releases in the Commercial Centre, the first monitoring was conducted after 4 egg releases, after 8 weeks on 15 January 2018.

Two days prior to the initial releases of *Wolbachia*-carrying *Ae*. *aegypti* in each site, fogging to suppress the wild populations was carried out using ultra low volume spray (ULV) of pyrethroids or organophosphates, in accordance with the Standard Operative Procedures of the Ministry of Health. During the trial release period, no fogging or space spraying activities were conducted within the release sites. GIS maps of release sites were prepared in order to construct release point grids, using the GIS software ArcGIS v10.5 and QGIS 3.2.3 Bonn. Release site maps were sketched based on the topography and cadastral map provided by the Department of Survey and Mapping Malaysia and with reference to Google Maps and 1MalaysiaMap (http://1malaysiamap.mygeoportal.gov.my/).

Mosquitoes were transported to the field sites in a van kept at ambient temperature. At Mentari Court, a total of 40,800 *Wolbachia*-carrying adult mosquitoes were released weekly in blocks A to G including indoor parking areas. The mosquitoes were released on every third level in each block at 4 release points, except for block G which had 6 release points. For the indoor parking building, there were 4 release points on the ground floor and 2nd floor. Four paper cups (diameter 8 cm, height 11 cm), each with 50 mosquitoes (mixed sex) were released. Following each release 10 containers were brought back to the laboratory and survival of adults was monitored; very little mortality (never > 5%) was observed. Releases were conducted for 20 weeks and terminated when the *Wolbachia* frequency reached > 90% on 3 consecutive monitoring periods. At the final monitoring period 98% were *Wolbachia*-carrying, as assessed through ovitraps. At Section 7 Flats, 20,400 *Wolbachia*-carrying adults were released weekly in blocks 1 to 51. There were 8 release points across 2 levels (ground floor and 2nd floor), with 1 cup per release point. Releases were initially conducted across 26 weeks when *Wolbachia* frequencies exceeded > 90% on 3 consecutive monitoring periods. Releases ceased for 4 months, and then a second release period was undertaken for 31 weeks at a lower rate (10,200).

At the Section 7 Commercial Centre, releases involved eggs rather than adults. Egg containers (14.5 cm x 12.5 cm) contained 150 *Wolbachia*-carrying eggs in 400 mL of water with 180 mg liver powder. A single release hole (2 cm) was covered with a stopper. Egg

containers were placed at a release point in the shade, on mailboxes or near staircases. Containers were left for 2 weeks. After 5 days, the stopper was removed to allow mosquitoes to emerge. Hatching rate was assessed on 5 containers by placing them in a sealed container left in the field house. Adults were counted (75%–80% of the eggs produced adults).

Monitoring mosquito population size and *Wolbachia* frequency

Ovitraps were used to assess *Wolbachia* frequencies and to monitor numbers of *Ae. aegypti* and *Ae. albopictus*. Each ovitrap consisted of a plastic container (96 mm height, 67 mm diameter) with 150 mL water and a wooden paddle (2 cm x 7 cm). In Mentari Court, 100 ovitraps were set up on the ground. In the apartment buildings, these were placed on the ground floor, the 2nd, 5th, 8th, 11th, 14th and 17th floors. For the car park building, the 1st floor and 3rd floors were monitored. Ovitraps were collected after a week and the paddle+water was transferred to a plastic container (12 × 12 cm). All emerging mosquitoes were identified to species and a maximum of 10 *Ae. aegypti* per trap were used for *Wolbachia* screening.

In Section 7 Shah Alam, monitoring was done after 4 weeks from the first release using 183 ovitraps, with 3 ovitraps set up per block on the ground, 3rd and 5th floors. Initially, monitoring in Mentari Court and Section 7 Shah Alam was conducted every two weeks and later every month. In Section 7 Commercial Centre, 100 ovitraps were set on the ground, middle and top floors as evenly as possible across the release zone. Monitoring was initiated after the 4th egg release on a monthly basis.

Adults from ovitraps were stored in absolute ethanol at −80°C. DNA was extracted from individual mosquitoes using the Chelex 100 resin (Bio-Rad Laboratories) method. Mosquitoes were homogenized in 175 μL of 5% Chelex solution using TissueLyser II machine (QIAGEN) and with 5 μL of proteinase K (20 mg/mL) (Bioron Life Science). The extraction was incubated in thermocycler at 65°C for 1 h, followed by incubation for 10 min at 90°C[16]. *Wolbachia* was detected by high-resolution melting polymerase chain reaction (qPCR-HRM) [27] with 1:10 diluted DNA using the following *wAlbB1*-specifc primers: *wAlbB1*-F (5′-CCTTACCTCCTGCACAACAA) and *wAlbB1*-R (5′ – GGATTGTCCAGTGGCCTTA), as well as universal mosquito primers: *mRpS6*_F (5′-AGTTGAACGTATCGTTT CCCGCTAC) and *mRpS6*_R (5′-GAAGTGACGCAGCTTGTGGTCGTCC), which target the conserved region of the *RpS6* gene, and *Ae*. *aegypti* primers *aRpS6*-F (5′-ATCAAGAAGCGCCGTGTCG) and *aRpS6*-R (5′-CAGGTGCAGGATCTTCATGTATTCG), which target the *Ae. aegypti*-specific polymorphisms within *RpS6* and do not amplify *Ae. albopictus*.

Reactions were run as 384-well plates in a LightCycler 480 II (Roche). qPCR-HRM was performed in 10 μL reactions containing 2 μL of DNA, 0.08 μL of 50 μM forward+reverse primer, 2.92 μL Milli-Q water and 5 μL Ronald’s Real-Time Buffer (3.28 μL Milli-Q water, 0.4 μL $MgCl_2$ (50 mM), 1.0 μL ThermoPol Reaction Buffer with 20 mM Magnesium (10x), 0.25 μL HRM Master (Roche), 0.064 μL dNTPs (25 mM) and 0.01 μL Immolase (20 U/μL). qPCR was run following cycling conditions: 95°C for 10 min, followed by 50 cycles of 95°C for 10 s, 58°C for 15 s, 72°C for 15 s. High resolution melting was performed by heating the PCR product to 95°C, and then cooling to 40°C. Then the temperature was increased to 65°C. Samples were considered positive for *Wolbachia* when the Tm for the amplicon produced by the *Ae. aegypti* primers was at least 84°C and the Tm for the *Wolbachia*-primer amplicon was around 80°C.

Dengue control activities

In Mentari Court, many different control activities and education programmes have been carried out to suppress dengue. Figure 5 shows that various activities such as source reduction, thermal fogging and Ultra Low Volume space spraying were conducted. Despite this activity, dengue cases remained high in this site. After the introduction of *Wolbachia,* there was a decrease in other activities undertaken to suppress dengue following a decrease in cases. Therefore, community engagement around *Wolbachia* decreased rather than increased other control activities at this site. At the other release sites, there was no increase in non-*Wolbachia* dengue suppression activities following the initiation of releases.

Dengue incidence

Matched control sites were selected based on similarity of constituent buildings (at least 7 blocks of 12-20 floors for Mentari Court controls, at least 35 blocks of 4-7 floors for Shah Alam Flat controls, at least 15 blocks of 5-7 floors for Commercial Centre controls); and comparable dengue incidence between 2013 and the start of intervention periods. Notified dengue cases in release and control sites were recorded daily from local clinics and hospitals. All notified cases were confirmed using NS1 and IgM/IgG Combo Rapid Test Kits (RVR Diagnostics Sdn. Bhd., Subang Jaya, Malaysia) according to manufacturer’s protocols, based on the established Malaysian Dengue Clinical Practice Guidelines (CPG), available on http://www.moh.gov.my.

All notified dengue cases are recorded into the National e-dengue database at the District Health Office. Dengue cases in the population residing at the study sites were detected via this National Dengue Surveillance System. All cases were diagnosed using the National case definition guidelines (Case Definitions for Infectious Diseases in Malaysia 2017). A confirmed case of dengue was defined as fulfilling the clinical criteria for dengue infection with the following laboratory confirmation: detection of Dengue Non-Structural Protein 1 (NS1) from serum; and, detection of dengue IgM and /or IgG from a single serum sample. While there are limitations to all laboratory diagnostic tests for dengue, the dengue rapid test kits (RTK) applied in this National Surveillance System represent the most cost-effective point of care diagnostic testing at a population level. The standardized diagnostic criteria applied through this system ensures no biases in test results between the intervention and control sites.

QUANTIFICATION AND STATISTICAL ANALYSIS

A Bayesian time series model was used to estimate the reduction in dengue cases resulting from releases. The model structure was as follows:

$$y_{i,t} \sim Poisson(\lambda_{i,t} N_i)$$

$$ln(\lambda_{i,t}) = \alpha_i + \gamma_{i,t} + \beta x_i$$

$$\gamma_{i,t} = \rho \gamma_{i,t-1} + \epsilon_{i,t};\ \gamma_{i,0} = 0$$

$$r \sim N^+(0, 100);\ \epsilon_{i,t} \sim N(0, \sigma^2);\ \rho \sim U(-1, 1);\ \sigma \sim N^+(0, 100);\ \alpha_i \sim N(0, 100)$$

where the number of cases y at each site i and week t were assumed to follow a Poisson distribution, with the expected count given by the product of population at that site, and the per-capita incidence which varied varying through time and between sites. Each site had a separate time series of log-incidences $\alpha_i + \gamma_{i,t}$ with temporal correlation driven by an autoregressive model of order one, with parameters ρ and σ^2 shared by all sites. Each observation therefore had a separate (temporally-correlated) random effect on the log scale, to account for extra-Poisson dispersion and temporal correlation. The intervention effect was represented by a parameter β and an indicator variable x_i for whether the observation was post-release at a release site. Model parameters were assigned vague normal; positive-truncated normal; or uniform priors. The model was used both to estimate the impact of the releases, and to assess the evidence from the dengue case data that releases lead to a reduction in incidence - quantified as the posterior probability that β is negative, in the absence of prior knowledge about the direction of the effect.

Posterior samples of model parameters were simulated by Hamiltonian Monte Carlo in greta [29] with 4 chains, each yielding 4000 posterior samples of model parameters after a warmup period of 1000 iterations during which period the leapfrog step size and diagonal mass matrix parameters were tuned. The number of leapfrog steps was sampled uniformly from between 30 and 40 throughout. Convergence was assessed by the Gelman-Rubin $\widehat{R}$ diagnostic, using the coda R package [30] ($\widehat{R} \leq 1.01$ for all parameters) and visual assessment of trace plots. Model fit was assessed by posterior predictive simulation: a random dataset of $y_{i,t}$ values was generated according to each posterior sample of $p_{i,t}$ and r, and the distributions of the simulated $y_{i,t}$ values were compared with the observed $y_{i,t}$.

DATA AND CODE AVAILABILITY

The datasets generated during this study are available at Mendeley Data https://doi.org/10.17632/v8vn35zj3g.1. The analysis code is freely available online https://doi.org/10.5281/zenodo.3520216.

Current Biology, Volume *29*

Supplemental Information

Establishment of *Wolbachia* Strain *w*AlbB in Malaysian Populations of *Aedes aegypti* for Dengue Control

Wasi A. Nazni, Ary A. Hoffmann, Ahmad NoorAfizah, Yoon Ling Cheong, Maria V. Mancini, Nicholas Golding, Ghazali M.R. Kamarul, Mohd A.K. Arif, Hasim Thohir, Halim NurSyamimi, M. Zabari ZatilAqmar, Mazni NurRuqqayah, Amran NorSyazwani, Azmi Faiz, Francis-Rudin M.N. Irfan, Subramaniam Rubaaini, Nasir Nuradila, Nasir M.N. Nizam, Saidin M. Irwan, Nancy M. Endersby-Harshman, Vanessa L. White, Thomas H. Ant, Christie S. Herd, Asim H. Hasnor, Rahman AbuBakar, Dusa M. Hapsah, Khairuddin Khadijah, Denim Kamilan, Soo Cheng Lee, Yusof M. Paid, Kamaludin Fadzilah, Omar Topek, Balvinder S. Gill, Han Lim Lee, and Steven P. Sinkins

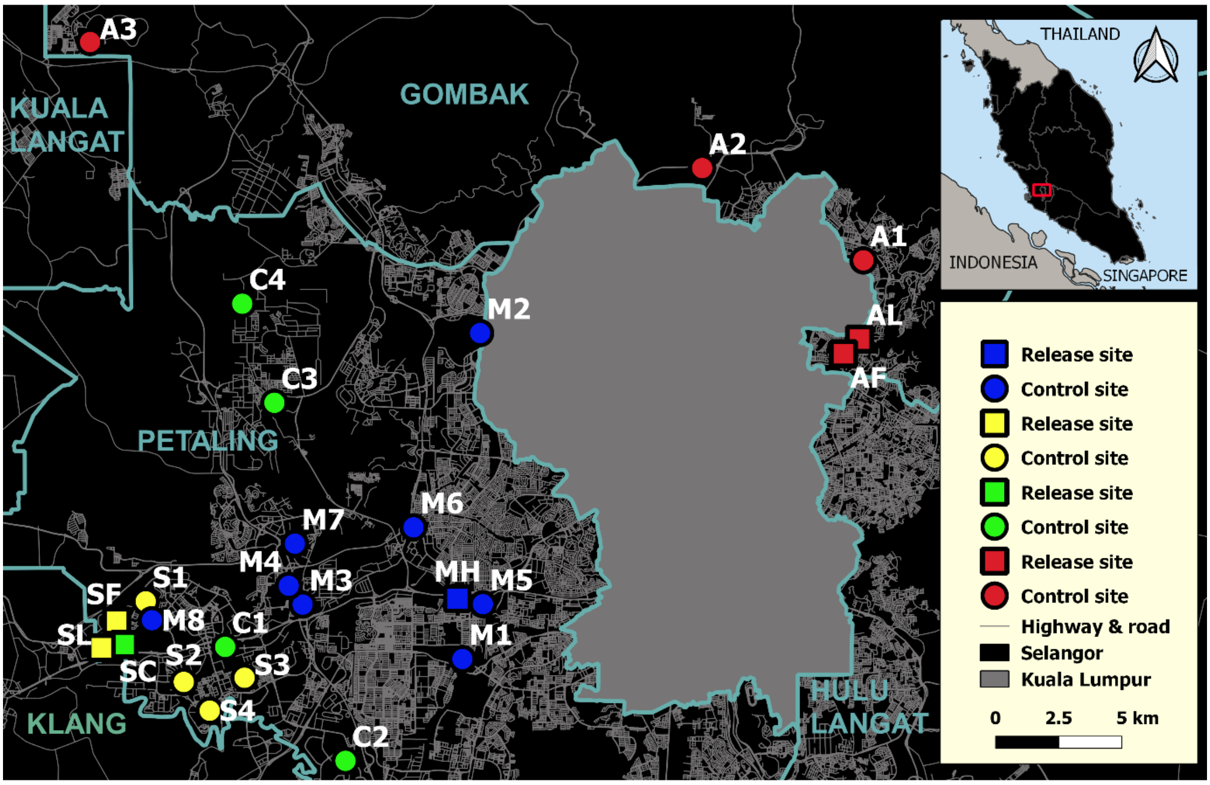

Figure S1. Maps of all six release zones and controls in the Petaling and Gombak Districts of Selangor, Related to Figure 1. Mentari Court high rise (MH) and controls (M1-M8); S7 Flats & Landed (SF & SL) and controls (S1-S4); S7 Commercial (SC) and controls (C1-C4); AU2 landed & AU2 PKNS Flats (AL & AF) and controls (A1-3). Table 1 contains additional details about release and controls sites. Map constructed using MapCruzin.com, OpenStreetMap.org under the Open Database License (http://opendatacommons.org/licenses/odbl/1.0/)).

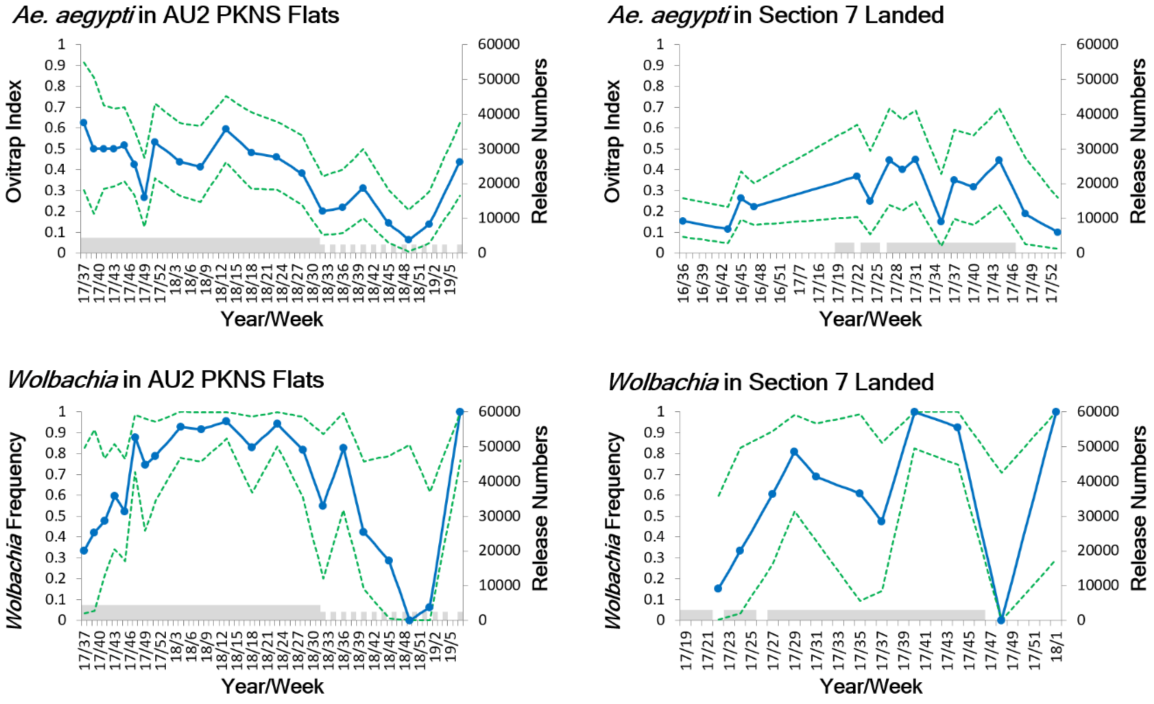

Figure S2. ***Aedes aegypti* population size and *Wolbachia* frequency estimates for two smaller secondary release sites, Related to Figures 2 and 3.** Population size was measured through an ovitrap index (*Ae. aegypti*- positive traps divided by total number of traps) during the release/monitoring period. Grey shaded areas represent release periods; 95% confidence intervals are shown as dotted lines.

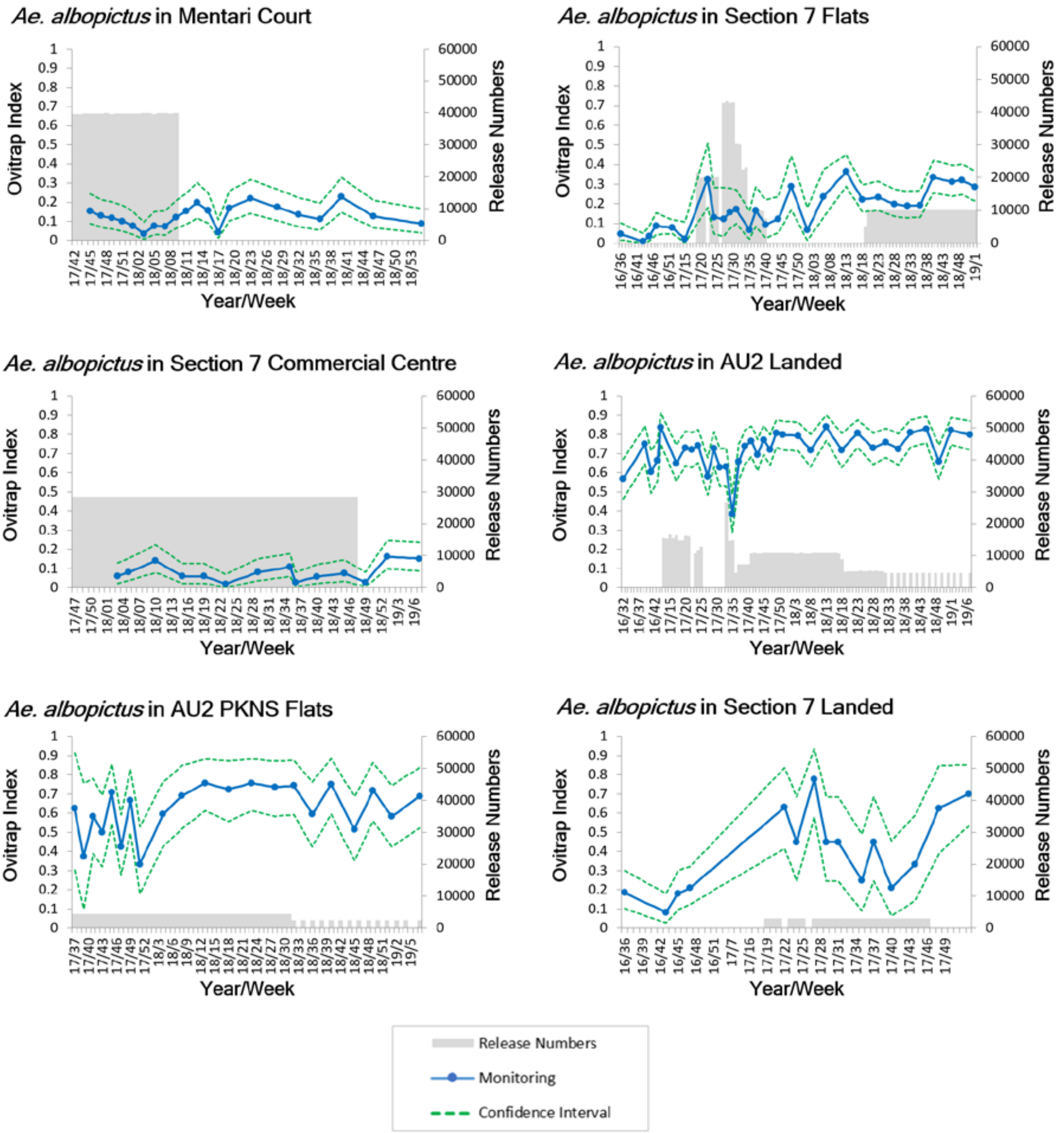

Figure S3. ***Aedes albopictus* population size estimates at release sites, related to figure 2.** Size was measured through an ovitrap index (*Ae. albopictus*-positive traps divided by total number of traps) during the release/monitoring period. Grey shaded areas represent release periods; 95% confidence intervals are shown as dotted lines.

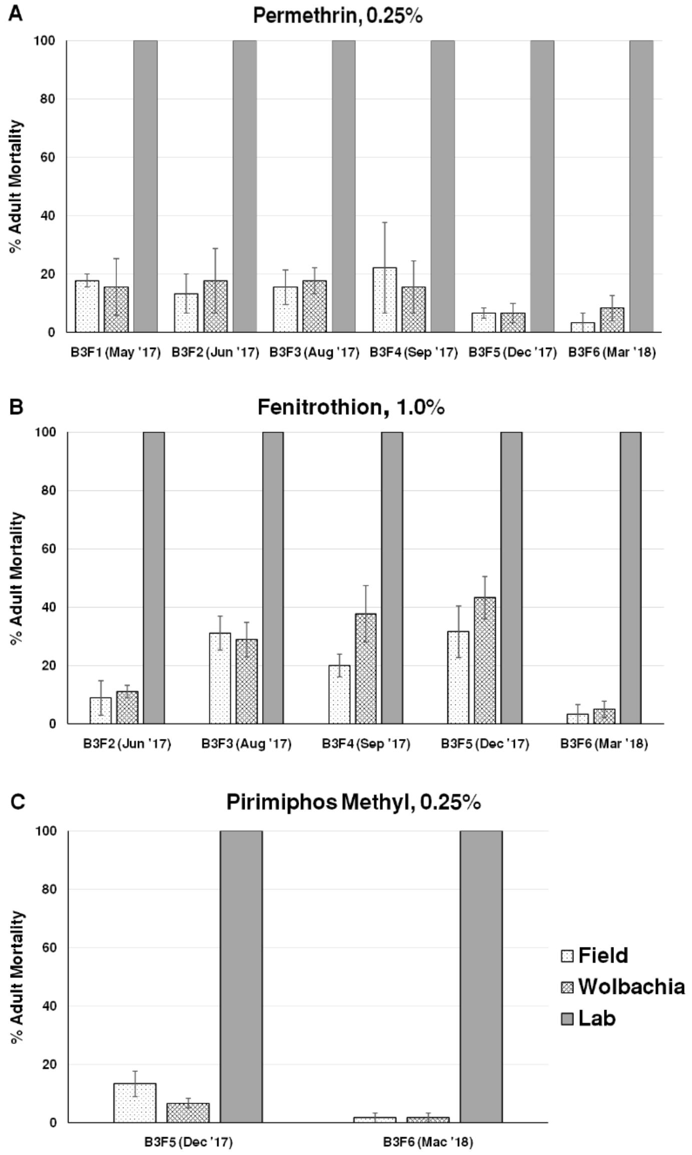

Figure S4. Insecticide susceptibility of the release line ("Wolbachia") compared with the field-derived wild-type population of *Ae. aegypti* ("Field") and a laboratory insecticide susceptible line ("Lab"), related to STAR Methods. Lines were tested against three pesticides: permethrin, fenitrothion, and pirimiphos methyl. Bioassays measured adult mortality at discriminating doses. Error bars represent standard errors.

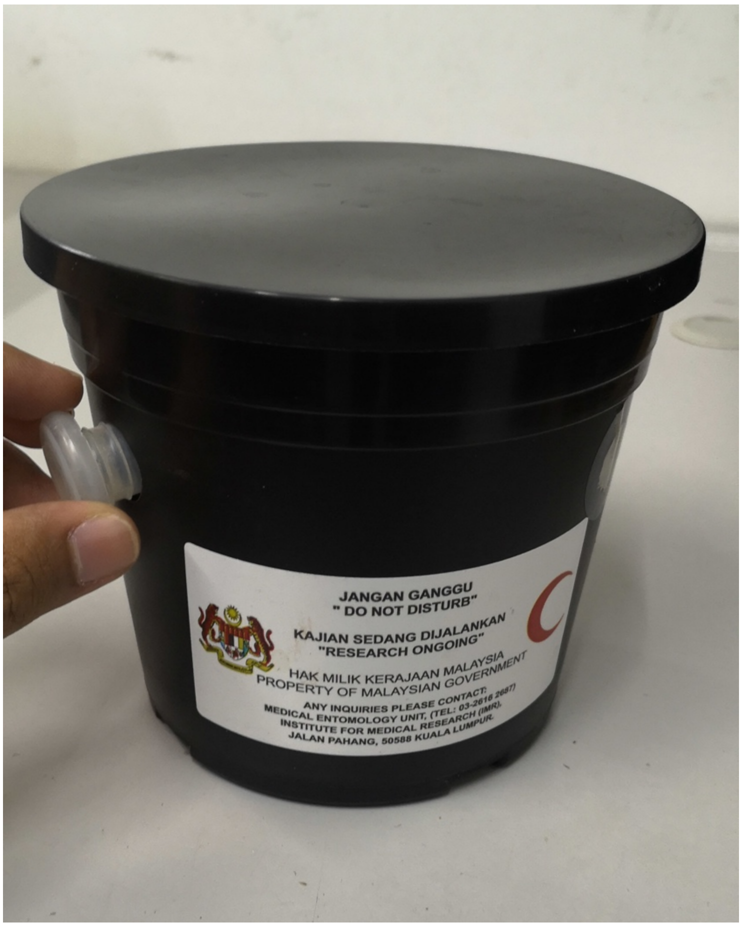

Figure S5. Example of an egg release container as used in the Section 7 Commercial Centre site, related to Figure 3. Note the 2 cm diameter release holes and plug, removed to allow escape of adult mosquitoes.

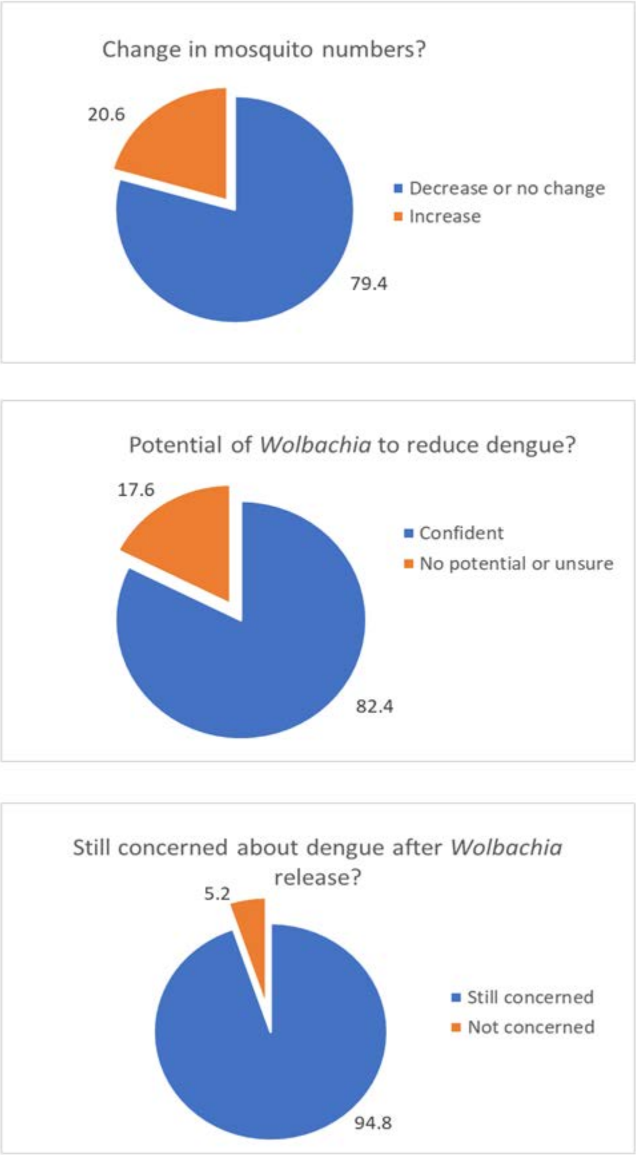

Figure S6. Post-release survey questionnaire results from community engagement activities in Section 7 Flats, related to figures 2 and 3. Data were collected from a door-to-door survey in the Section 7 Flats release area 2 months after the beginning of the releases. 196 respondents were included, consisting of 40% females, with age classes 29-39 (15%), 40-50 (65%) and >60 (10%).

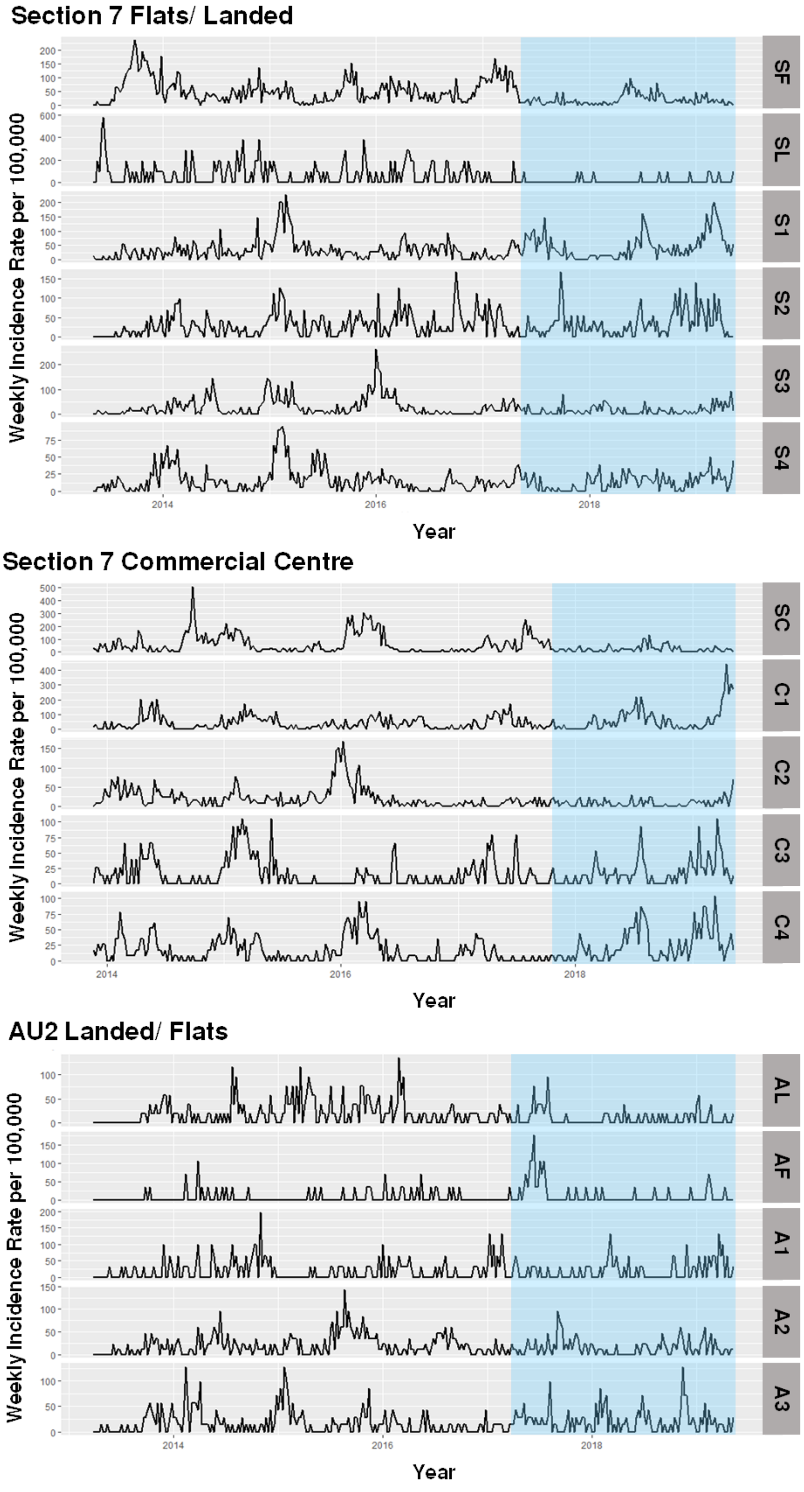

Figure S7. Dengue incidence plots at the Shah Alam Section 7 PKNS flats & Landed, Section 7 Commercial Centre, and AU2 Landed / Flats release and matched control sites, related to Figure 4. SF, SL, SC, AL and AF represents release areas on different sites. See Table 1 for additional details on control sites. The periods during and after commencement of *Wolbachia* releases are indicated by blue regions.
